# Supplementary material for: Synaptotagmin 7 is targeted to the axonal plasma membrane through γ-secretase processing to promote synaptic vesicle docking in mouse hippocampal neurons
Source: eLife. 2021 Sep 20;10:e67261. doi: 10.7554/eLife.67261 (PMC8452306; doi:10.7554/eLife.67261)
Supplement: Figure 1—source data 1. [file elife-67261-fig1-data1.docx]

**Figure 1e – source data 1**

| Compare each cell mean with the other cell mean in that row | | |  |  |  |  |  |
| --- | --- | --- | --- | --- | --- | --- | --- |
|  |  |  |  |  |  |  |  |
| Number of families | 1 |  |  |  |  |  |  |
| Number of comparisons per family | 4 |  |  |  |  |  |  |
| Alpha | 0.05 |  |  |  |  |  |  |
|  |  |  |  |  |  |  |  |
| Sidak's multiple comparisons test | Predicted (LS) mean diff, | 95,00% CI of diff, | Significant? | Summary | Adjusted P Value | |  |
|  |  |  |  |  |  |  |  |
| WT - S7KO |  |  |  |  |  |  |  |
| 20 Hz | 0.5768 | 0,3901 to 0,7634 | Yes | **** | <0,0001 |  |  |
| 10 Hz | 0.4322 | 0,2387 to 0,6256 | Yes | **** | <0,0001 |  |  |
| 5 Hz | 0.2763 | 0,08962 to 0,4629 | Yes | ** | 0.0012 |  |  |
| 2 Hz | 0.1031 | -0,09391 to 0,3001 | No | ns | 0.5643 |  |  |
|  |  |  |  |  |  |  |  |
|  |  |  |  |  |  |  |  |
| Test details | Predicted (LS) mean 1 | Predicted (LS) mean 2 | Predicted (LS) mean diff, | SE of diff, | N1 | N2 | t |
|  |  |  |  |  |  |  |  |
| WT - S7KO |  |  |  |  |  |  |  |
| 20 Hz | 1.153 | 0.5767 | 0.5768 | 0.07362 | 14 | 15 | 7.835 |
| 10 Hz | 0.9148 | 0.4827 | 0.4322 | 0.0763 | 14 | 13 | 5.664 |
| 5 Hz | 0.7609 | 0.4846 | 0.2763 | 0.07362 | 15 | 14 | 3.753 |
| 2 Hz | 0.7013 | 0.5982 | 0.1031 | 0.0777 | 13 | 13 | 1.327 |
|  |  |  |  |  |  |  |  |
|  | delta T (ms) | WT |  |  | S7KO |  |  |
|  | X | Mean | SEM | N | Mean | SEM | N |
| 20 Hz | 50 | 1.153452658 | 0.092200939 | 14 | 0.576676482 | 0.029094085 | 15 |
| 10 Hz | 100 | 0.914810555 | 0.069095618 | 14 | 0.482658414 | 0.027627979 | 13 |
| 5 Hz | 200 | 0.760889287 | 0.045842498 | 15 | 0.484637944 | 0.038067777 | 14 |
| 2 Hz | 500 | 0.701312358 | 0.039677713 | 13 | 0.598230091 | 0.049256498 | 13 |
