# Supplementary material for: Synaptotagmin 7 is targeted to the axonal plasma membrane through γ-secretase processing to promote synaptic vesicle docking in mouse hippocampal neurons
Source: eLife. 2021 Sep 20;10:e67261. doi: 10.7554/eLife.67261 (PMC8452306; doi:10.7554/eLife.67261)
Supplement: Figure 1—source data 2. [file elife-67261-fig1-data2.docx]

**Figure 1f – source data 2**

| Compare each cell mean with the other cell mean in that row | | | |  |  |  |  |  |
| --- | --- | --- | --- | --- | --- | --- | --- | --- |
|  |  |  |  |  |  |  |  |  |
| Number of families | 1 |  |  |  |  |  |  |  |
| Number of comparisons per family | 4 |  |  |  |  |  |  |  |
| Alpha | 0.05 |  |  |  |  |  |  |  |
|  |  |  |  |  |  |  |  |  |
| Sidak's multiple comparisons test | Predicted (LS) mean diff, | 95,00% CI of diff, | Significant? | Summary | Adjusted P Value |  |  |  |
|  |  |  |  |  |  |  |  |  |
| WT - S7KO |  |  |  |  |  |  |  |  |
| 20 Hz | 0.1668 | 0,03881 to 0,2949 | Yes | ** | 0.0052 |  |  |  |
| 10 Hz | 0.1593 | 0,02908 to 0,2895 | Yes | ** | 0.0099 |  |  |  |
| 5 Hz | 0.1382 | 0,01017 to 0,2662 | Yes | * | 0.0289 |  |  |  |
| 2 Hz | 0.04023 | -0,09248 to 0,1729 | No | ns | 0.9045 |  |  |  |
|  |  |  |  |  |  |  |  |  |
|  |  |  |  |  |  |  |  |  |
| Test details | Predicted (LS) mean 1 | Predicted (LS) mean 2 | Predicted (LS) mean diff, | SE of diff, | N1 | N2 | t | DF |
|  |  |  |  |  |  |  |  |  |
| WT - S7KO |  |  |  |  |  |  |  |  |
| 20 Hz | 1.034 | 0.867 | 0.1668 | 0.05052 | 14 | 15 | 3.302 | 105 |
| 10 Hz | 0.9919 | 0.8326 | 0.1593 | 0.05139 | 14 | 14 | 3.1 | 105 |
| 5 Hz | 0.9626 | 0.8244 | 0.1382 | 0.05052 | 15 | 14 | 2.736 | 105 |
| 2 Hz | 0.9353 | 0.8951 | 0.04023 | 0.05236 | 13 | 14 | 0.7682 | 105 |
|  |  |  |  |  |  |  |  |  |
|  | delta T (ms) | WT |  |  | S7KO |  |  |  |
|  | X | Mean | SEM | N | Mean | SEM | N |  |
| 20 Hz | 50 | 1.033892857 | 0.042603632 | 14 | 0.867046667 | 0.033094452 | 15 |  |
| 10 Hz | 100 | 0.991928571 | 0.025469185 | 14 | 0.832621429 | 0.045387828 | 14 |  |
| 5 Hz | 200 | 0.962633333 | 0.015332967 | 15 | 0.824428571 | 0.04806574 | 14 |  |
| 2 Hz | 500 | 0.935276923 | 0.027039859 | 13 | 0.89505 | 0.040833811 | 14 |  |
