# Supplementary material for: Synaptotagmin 7 is targeted to the axonal plasma membrane through γ-secretase processing to promote synaptic vesicle docking in mouse hippocampal neurons
Source: eLife. 2021 Sep 20;10:e67261. doi: 10.7554/eLife.67261 (PMC8452306; doi:10.7554/eLife.67261)
Supplement: Figure 1—figure supplement 1—source data 1. [file elife-67261-fig1-figsupp1-data1.docx]

**Figure 1 – figure supplement 1b – source data 1**

| Compare each cell mean with the other cell mean in that row | | | | |  |  |  |  |
| --- | --- | --- | --- | --- | --- | --- | --- | --- |
|  |  |  |  |  |  |  |  |  |
| Number of families | 1 |  |  |  |  |  |  |  |
| Number of comparisons per family | 4 |  |  |  |  |  |  |  |
| Alpha | 0.05 |  |  |  |  |  |  |  |
|  |  |  |  |  |  |  |  |  |
| Sidak's multiple comparisons test | Predicted (LS) mean diff, | 95,00% CI of diff, | Significant? | Summary | Adjusted P Value | |  |  |
|  |  |  |  |  |  |  |  |  |
| WT - S7KO |  |  |  |  |  |  |  |  |
| 20 Hz | 0.102 | 0,06380 to 0,1401 | Yes | **** | <0,0001 |  |  |  |
| 10 Hz | 0.1004 | 0,06157 to 0,1392 | Yes | **** | <0,0001 |  |  |  |
| 5 Hz | 0.06116 | 0,02301 to 0,09932 | Yes | *** | 0.0004 |  |  |  |
| 2 Hz | 0.01736 | -0,02219 to 0,05691 | No | ns | 0.7137 |  |  |  |
|  |  |  |  |  |  |  |  |  |
|  |  |  |  |  |  |  |  |  |
| Test details | Predicted (LS) mean 1 | Predicted (LS) mean 2 | Predicted (LS) mean diff, | SE of diff, | N1 | N2 | t | DF |
|  |  |  |  |  |  |  |  |  |
| WT - S7KO |  |  |  |  |  |  |  |  |
| 20 Hz | 0.3037 | 0.2018 | 0.102 | 0.01506 | 14 | 15 | 6.772 | 105 |
| 10 Hz | 0.2897 | 0.1893 | 0.1004 | 0.01531 | 14 | 14 | 6.555 | 105 |
| 5 Hz | 0.2497 | 0.1885 | 0.06116 | 0.01506 | 15 | 14 | 4.062 | 105 |
| 2 Hz | 0.2231 | 0.2058 | 0.01736 | 0.01561 | 13 | 14 | 1.112 | 105 |
