# Supplementary material for: Synaptotagmin 7 is targeted to the axonal plasma membrane through γ-secretase processing to promote synaptic vesicle docking in mouse hippocampal neurons
Source: eLife. 2021 Sep 20;10:e67261. doi: 10.7554/eLife.67261 (PMC8452306; doi:10.7554/eLife.67261)
Supplement: Figure 3—source data 1. [file elife-67261-fig3-data1.docx]

**Figure 3c – source data 4**

| Number of families | 1 |  |  |  |  |  |
| --- | --- | --- | --- | --- | --- | --- |
| Number of comparisons per family | 16 |  |  |  |  |  |
| Alpha | 0.05 |  |  |  |  |  |
|  |  |  |  |  |  |  |
| Dunn's multiple comparisons test | Mean rank diff. | Significant? | Summary | Adjusted P Value | |  |
| WT no stim vs. S7KO no stim | 9.714 | No | ns | >0.9999 | A-E |  |
| WT 5 ms SS vs. S7KO 5 ms SS | 259.7 | Yes | *** | 0.0001 | B-F |  |
| WT 5 ms 50 AP 20Hz vs. S7KO 5 ms 50 AP 20Hz | 338 | Yes | **** | <0.0001 | C-G |  |
| WT 5 s 50 AP 20Hz vs. S7KO 5 s 50 AP 20Hz | 258.1 | Yes | **** | <0.0001 | D-H |  |
| WT no stim vs. WT 5 ms SS | 498.2 | Yes | **** | <0.0001 | A-B |  |
| WT no stim vs. WT 5 ms 50 AP 20Hz | 444 | Yes | **** | <0.0001 | A-C |  |
| WT no stim vs. WT 5 s 50 AP 20Hz | 270.1 | Yes | **** | <0.0001 | A-D |  |
| WT 5 ms SS vs. WT 5 ms 50 AP 20Hz | -54.2 | No | ns | >0.9999 | B-C |  |
| WT 5 ms SS vs. WT 5 s 50 AP 20Hz | -228 | Yes | ** | 0.0011 | B-D |  |
| WT 5 ms 50 AP 20Hz vs. WT 5 s 50 AP 20Hz | -173.8 | Yes | * | 0.0343 | C-D |  |
| S7KO no stim vs. S7KO 5 ms SS | 748.2 | Yes | **** | <0.0001 | E-F |  |
| S7KO no stim vs. S7KO 5 ms 50 AP 20Hz | 772.2 | Yes | **** | <0.0001 | E-G |  |
| S7KO no stim vs. S7KO 5 s 50 AP 20Hz | 518.5 | Yes | **** | <0.0001 | E-H |  |
| S7KO 5 ms SS vs. S7KO 5 ms 50 AP 20Hz | 24.09 | No | ns | >0.9999 | F-G |  |
| S7KO 5 ms SS vs. S7KO 5 s 50 AP 20Hz | -229.7 | Yes | *** | 0.0009 | F-H |  |
| S7KO 5 ms 50 AP 20Hz vs. S7KO 5 s 50 AP 20Hz | -253.8 | Yes | *** | 0.0003 | G-H |  |
|  |  |  |  |  |  |  |
| Test details | Mean rank 1 | Mean rank 2 | Mean rank diff. | n1 | n2 | Z |
| WT no stim vs. S7KO no stim | 1684 | 1674 | 9.714 | 323 | 307 | 0.166 |
| WT 5 ms SS vs. S7KO 5 ms SS | 1186 | 926.2 | 259.7 | 322 | 319 | 4.478 |
| WT 5 ms 50 AP 20Hz vs. S7KO 5 ms 50 AP 20Hz | 1240 | 902.1 | 338 | 338 | 271 | 5.647 |
| WT 5 s 50 AP 20Hz vs. S7KO 5 s 50 AP 20Hz | 1414 | 1156 | 258.1 | 334 | 341 | 4.567 |
| WT no stim vs. WT 5 ms SS | 1684 | 1186 | 498.2 | 323 | 322 | 8.618 |
| WT no stim vs. WT 5 ms 50 AP 20Hz | 1684 | 1240 | 444 | 323 | 338 | 7.773 |
| WT no stim vs. WT 5 s 50 AP 20Hz | 1684 | 1414 | 270.1 | 323 | 334 | 4.715 |
| WT 5 ms SS vs. WT 5 ms 50 AP 20Hz | 1186 | 1240 | -54.2 | 322 | 338 | 0.9482 |
| WT 5 ms SS vs. WT 5 s 50 AP 20Hz | 1186 | 1414 | -228 | 322 | 334 | 3.978 |
| WT 5 ms 50 AP 20Hz vs. WT 5 s 50 AP 20Hz | 1240 | 1414 | -173.8 | 338 | 334 | 3.07 |
| S7KO no stim vs. S7KO 5 ms SS | 1674 | 926.2 | 748.2 | 307 | 319 | 12.75 |
| S7KO no stim vs. S7KO 5 ms 50 AP 20Hz | 1674 | 902.1 | 772.2 | 307 | 271 | 12.62 |
| S7KO no stim vs. S7KO 5 s 50 AP 20Hz | 1674 | 1156 | 518.5 | 307 | 341 | 8.978 |
| S7KO 5 ms SS vs. S7KO 5 ms 50 AP 20Hz | 926.2 | 902.1 | 24.09 | 319 | 271 | 0.3973 |
| S7KO 5 ms SS vs. S7KO 5 s 50 AP 20Hz | 926.2 | 1156 | -229.7 | 319 | 341 | 4.017 |
| S7KO 5 ms 50 AP 20Hz vs. S7KO 5 s 50 AP 20Hz | 902.1 | 1156 | -253.8 | 271 | 341 | 4.248 |
| S7KO 5 ms 50 AP 20Hz vs. S7KO 5 s 50 AP 20Hz | 902.1 | 1156 | -253.8 | 271 | 341 | 4.248 |
