# Supplementary material for: Synaptotagmin 7 is targeted to the axonal plasma membrane through γ-secretase processing to promote synaptic vesicle docking in mouse hippocampal neurons
Source: eLife. 2021 Sep 20;10:e67261. doi: 10.7554/eLife.67261 (PMC8452306; doi:10.7554/eLife.67261)
Supplement: Figure 3—source data 2. [file elife-67261-fig3-data2.docx]

**Figure 3d – source data 5**

| Number of families | 1 |  |  |  |  |  |
| --- | --- | --- | --- | --- | --- | --- |
| Number of comparisons per family | 16 |  |  |  |  |  |
| Alpha | 0.05 |  |  |  |  |  |
|  |  |  |  |  |  |  |
| Dunn's multiple comparisons test | Mean rank diff. | Significant? | Summary | Adjusted P Value | |  |
| WT no stim vs. WT 5 ms SS | 89.61 | No | ns | >0.9999 | A-B |  |
| WT no stim vs. WT 5 ms 50 AP 20Hz | 276.4 | Yes | **** | <0.0001 | A-C |  |
| WT no stim vs. WT 5 s 50 AP 20Hz | 18.8 | No | ns | >0.9999 | A-D |  |
| WT 5 ms SS vs. WT 5 s 50 AP 20Hz | -70.81 | No | ns | >0.9999 | B-D |  |
| WT 5 ms 50 AP 20Hz vs. WT 5 s 50 AP 20Hz | -257.6 | Yes | **** | <0.0001 | C-D |  |
| S7KO no stim vs. S7KO 5 ms SS | 169.4 | No | ns | 0.066 | E-F |  |
| S7KO no stim vs. S7KO 5 ms 50 AP 20Hz | 424.3 | Yes | **** | <0.0001 | E-G |  |
| S7KO no stim vs. S7KO 5 s 50 AP 20Hz | 269 | Yes | **** | <0.0001 | E-H |  |
| S7KO 5 ms SS vs. S7KO 5 s 50 AP 20Hz | 99.55 | No | ns | >0.9999 | F-H |  |
| S7KO 5 ms 50 AP 20Hz vs. S7KO 5 s 50 AP 20Hz | -155.3 | No | ns | 0.1567 | G-H |  |
| S7KO 5 ms SS vs. S7KO 5 ms 50 AP 20Hz | 254.9 | Yes | *** | 0.0005 | F-G |  |
| WT no stim vs. S7KO no stim | -7.918 | No | ns | >0.9999 | A-E |  |
| WT 5 ms SS vs. S7KO 5 ms SS | 71.89 | No | ns | >0.9999 | B-F |  |
| WT 5 ms 50 AP 20Hz vs. S7KO 5 ms 50 AP 20Hz | 139.9 | No | ns | 0.3218 | C-G |  |
| WT 5 s 50 AP 20Hz vs. S7KO 5 s 50 AP 20Hz | 242.3 | Yes | *** | 0.0003 | D-H |  |
| WT 5 ms SS vs. WT 5 ms 50 AP 20Hz | 186.8 | Yes | * | 0.0183 | B-C |  |
|  |  |  |  |  |  |  |
| Test details | Mean rank 1 | Mean rank 2 | Mean rank diff. | n1 | n2 | Z |
| WT no stim vs. WT 5 ms SS | 1428 | 1339 | 89.61 | 324 | 323 | 1.543 |
| WT no stim vs. WT 5 ms 50 AP 20Hz | 1428 | 1152 | 276.4 | 324 | 339 | 4.816 |
| WT no stim vs. WT 5 s 50 AP 20Hz | 1428 | 1410 | 18.8 | 324 | 335 | 0.3265 |
| WT 5 ms SS vs. WT 5 s 50 AP 20Hz | 1339 | 1410 | -70.81 | 323 | 335 | 1.229 |
| WT 5 ms 50 AP 20Hz vs. WT 5 s 50 AP 20Hz | 1152 | 1410 | -257.6 | 339 | 335 | 4.526 |
| S7KO no stim vs. S7KO 5 ms SS | 1436 | 1267 | 169.4 | 307 | 319 | 2.868 |
| S7KO no stim vs. S7KO 5 ms 50 AP 20Hz | 1436 | 1012 | 424.3 | 307 | 271 | 6.89 |
| S7KO no stim vs. S7KO 5 s 50 AP 20Hz | 1436 | 1167 | 269 | 307 | 341 | 4.627 |
| S7KO 5 ms SS vs. S7KO 5 s 50 AP 20Hz | 1267 | 1167 | 99.55 | 319 | 341 | 1.73 |
| S7KO 5 ms 50 AP 20Hz vs. S7KO 5 s 50 AP 20Hz | 1012 | 1167 | -155.3 | 271 | 341 | 2.583 |
| S7KO 5 ms SS vs. S7KO 5 ms 50 AP 20Hz | 1267 | 1012 | 254.9 | 319 | 271 | 4.176 |
| WT no stim vs. S7KO no stim | 1428 | 1436 | -7.918 | 324 | 307 | 0.1346 |
| WT 5 ms SS vs. S7KO 5 ms SS | 1339 | 1267 | 71.89 | 323 | 319 | 1.233 |
| WT 5 ms 50 AP 20Hz vs. S7KO 5 ms 50 AP 20Hz | 1152 | 1012 | 139.9 | 339 | 271 | 2.324 |
| WT 5 s 50 AP 20Hz vs. S7KO 5 s 50 AP 20Hz | 1410 | 1167 | 242.3 | 335 | 341 | 4.263 |
| WT 5 ms SS vs. WT 5 ms 50 AP 20Hz | 1339 | 1152 | 186.8 | 323 | 339 | 3.252 |
