# Supplementary material for: Synaptotagmin 7 is targeted to the axonal plasma membrane through γ-secretase processing to promote synaptic vesicle docking in mouse hippocampal neurons
Source: eLife. 2021 Sep 20;10:e67261. doi: 10.7554/eLife.67261 (PMC8452306; doi:10.7554/eLife.67261)
Supplement: Figure 7—source data 1. [file elife-67261-fig7-data1.docx]

**Figure 7f – source data 6**

| Compare column means (main column effect) | | | |  |  |  |  |  |
| --- | --- | --- | --- | --- | --- | --- | --- | --- |
|  |  |  |  |  |  |  |  |  |
| Number of families | 1 |  |  |  |  |  |  |  |
| Number of comparisons per family | 15 |  |  |  |  |  |  |  |
| Alpha | 0.05 |  |  |  |  |  |  |  |
| Tukey's multiple comparisons test | Predicted (LS) mean diff, | 95,00% CI of diff, | Significant? | Summary | Adjusted P Value | |  |  |
|  |  |  |  |  |  |  |  |  |
| WT vs. S7KO (+CRE) | 0.2344 | 0,1995 to 0,2694 | Yes | **** | <0,0001 |  |  |  |
| WT vs. S7a Rescue | 0.07192 | 0,03824 to 0,1056 | Yes | **** | <0,0001 |  |  |  |
| WT vs. PM-S7a Rescue | 0.08996 | 0,05628 to 0,1236 | Yes | **** | <0,0001 |  |  |  |
| WT vs. Lyso-S7a Rescue | 0.05774 | 0,02405 to 0,09142 | Yes | **** | <0,0001 |  |  |  |
| WT vs. SV-S7a Rescue | 0.2143 | 0,1811 to 0,2474 | Yes | **** | <0,0001 |  |  |  |
| S7KO (+CRE) vs. S7a Rescue | -0.1625 | -0,1975 to -0,1275 | Yes | **** | <0,0001 |  |  |  |
| S7KO (+CRE) vs. PM-S7a Rescue | -0.1445 | -0,1794 to -0,1095 | Yes | **** | <0,0001 |  |  |  |
| S7KO (+CRE) vs. Lyso-S7a Rescue | -0.1767 | -0,2116 to -0,1417 | Yes | **** | <0,0001 |  |  |  |
| S7KO (+CRE) vs. SV-S7a Rescue | -0.02014 | -0,05458 to 0,01431 | No | ns | 0.554 |  |  |  |
| S7a Rescue vs. PM-S7a Rescue | 0.01804 | -0,01564 to 0,05172 | No | ns | 0.6467 |  |  |  |
| S7a Rescue vs. Lyso-S7a Rescue | -0.01418 | -0,04786 to 0,01950 | No | ns | 0.8368 |  |  |  |
| S7a Rescue vs. SV-S7a Rescue | 0.1424 | 0,1092 to 0,1755 | Yes | **** | <0,0001 |  |  |  |
| PM-S7a Rescue vs. Lyso-S7a Rescue | -0.03222 | -0,06590 to 0,001460 | No | ns | 0.0701 |  |  |  |
| PM-S7a Rescue vs. SV-S7a Rescue | 0.1243 | 0,09118 to 0,1575 | Yes | **** | <0,0001 |  |  |  |
| Lyso-S7a Rescue vs. SV-S7a Rescue | 0.1565 | 0,1234 to 0,1897 | Yes | **** | <0,0001 |  |  |  |
|  |  |  |  |  |  |  |  |  |
| Test details | Predicted (LS) mean 1 | Predicted (LS) mean 2 | Predicted (LS) mean diff, | SE of diff, | N1 | N2 | q | DF |
| WT vs. S7KO (+CRE) | 0.8053 | 0.5708 | 0.2344 | 0.01226 | 750 | 650 | 27.04 | 4150 |
| WT vs. S7a Rescue | 0.8053 | 0.7333 | 0.07192 | 0.01181 | 750 | 750 | 8.609 | 4150 |
| WT vs. PM-S7a Rescue | 0.8053 | 0.7153 | 0.08996 | 0.01181 | 750 | 750 | 10.77 | 4150 |
| WT vs. Lyso-S7a Rescue | 0.8053 | 0.7475 | 0.05774 | 0.01181 | 750 | 750 | 6.911 | 4150 |
| WT vs. SV-S7a Rescue | 0.8053 | 0.591 | 0.2143 | 0.01163 | 750 | 800 | 26.06 | 4150 |
| S7KO (+CRE) vs. S7a Rescue | 0.5708 | 0.7333 | -0.1625 | 0.01226 | 650 | 750 | 18.75 | 4150 |
| S7KO (+CRE) vs. PM-S7a Rescue | 0.5708 | 0.7153 | -0.1445 | 0.01226 | 650 | 750 | 16.66 | 4150 |
| S7KO (+CRE) vs. Lyso-S7a Rescue | 0.5708 | 0.7475 | -0.1767 | 0.01226 | 650 | 750 | 20.38 | 4150 |
| S7KO (+CRE) vs. SV-S7a Rescue | 0.5708 | 0.591 | -0.02014 | 0.01208 | 650 | 800 | 2.357 | 4150 |
| S7a Rescue vs. PM-S7a Rescue | 0.7333 | 0.7153 | 0.01804 | 0.01181 | 750 | 750 | 2.16 | 4150 |
| S7a Rescue vs. Lyso-S7a Rescue | 0.7333 | 0.7475 | -0.01418 | 0.01181 | 750 | 750 | 1.698 | 4150 |
| S7a Rescue vs. SV-S7a Rescue | 0.7333 | 0.591 | 0.1424 | 0.01163 | 750 | 800 | 17.32 | 4150 |
| PM-S7a Rescue vs. Lyso-S7a Rescue | 0.7153 | 0.7475 | -0.03222 | 0.01181 | 750 | 750 | 3.857 | 4150 |
| PM-S7a Rescue vs. SV-S7a Rescue | 0.7153 | 0.591 | 0.1243 | 0.01163 | 750 | 800 | 15.12 | 4150 |
| Lyso-S7a Rescue vs. SV-S7a Rescue | 0.7475 | 0.591 | 0.1565 | 0.01163 | 750 | 800 | 19.04 | 4150 |
