# Supplementary material for: Synaptotagmin 7 is targeted to the axonal plasma membrane through γ-secretase processing to promote synaptic vesicle docking in mouse hippocampal neurons
Source: eLife. 2021 Sep 20;10:e67261. doi: 10.7554/eLife.67261 (PMC8452306; doi:10.7554/eLife.67261)
Supplement: Figure 7—source data 2. [file elife-67261-fig7-data2.docx]

**Figure 7h – source data 7**

| Compare column means (main column effect) | | | |  |  |  |  |  |
| --- | --- | --- | --- | --- | --- | --- | --- | --- |
|  |  |  |  |  |  |  |  |  |
| Number of families | 1 |  |  |  |  |  |  |  |
| Number of comparisons per family | 15 |  |  |  |  |  |  |  |
| Alpha | 0.05 |  |  |  |  |  |  |  |
| Sidak's multiple comparisons test | Predicted (LS) mean diff, | 95,00% CI of diff, | Significant? | Summary | Adjusted P Value | |  |  |
|  |  |  |  |  |  |  |  |  |
| WT vs. S7KO (+CRE) | -0.03159 | -0,04868 to -0,01451 | Yes | **** | <0,0001 |  |  |  |
| WT vs. S7a Rescue | 0.007326 | -0,009140 to 0,02379 | No | ns | 0.9595 |  |  |  |
| WT vs. PM-S7a Rescue | -0.0004883 | -0,01697 to 0,01599 | No | ns | >0,9999 |  |  |  |
| WT vs. Lyso-S7a Rescue | -0.02815 | -0,04462 to -0,01169 | Yes | **** | <0,0001 |  |  |  |
| WT vs. SV-S7a Rescue | -0.05557 | -0,07177 to -0,03936 | Yes | **** | <0,0001 |  |  |  |
| S7KO (+CRE) vs. S7a Rescue | 0.03892 | 0,02183 to 0,05601 | Yes | **** | <0,0001 |  |  |  |
| S7KO (+CRE) vs. PM-S7a Rescue | 0.0311 | 0,01401 to 0,04820 | Yes | **** | <0,0001 |  |  |  |
| S7KO (+CRE) vs. Lyso-S7a Rescue | 0.003441 | -0,01365 to 0,02053 | No | ns | >0,9999 |  |  |  |
| S7KO (+CRE) vs. SV-S7a Rescue | -0.02397 | -0,04081 to -0,007134 | Yes | *** | 0.0005 |  |  |  |
| S7a Rescue vs. PM-S7a Rescue | -0.007814 | -0,02429 to 0,008664 | No | ns | 0.9329 |  |  |  |
| S7a Rescue vs. Lyso-S7a Rescue | -0.03548 | -0,05194 to -0,01901 | Yes | **** | <0,0001 |  |  |  |
| S7a Rescue vs. SV-S7a Rescue | -0.06289 | -0,07910 to -0,04668 | Yes | **** | <0,0001 |  |  |  |
| PM-S7a Rescue vs. Lyso-S7a Rescue | -0.02766 | -0,04414 to -0,01119 | Yes | **** | <0,0001 |  |  |  |
| PM-S7a Rescue vs. SV-S7a Rescue | -0.05508 | -0,07130 to -0,03886 | Yes | **** | <0,0001 |  |  |  |
| Lyso-S7a Rescue vs. SV-S7a Rescue | -0.02741 | -0,04362 to -0,01121 | Yes | **** | <0,0001 |  |  |  |
|  |  |  |  |  |  |  |  |  |
| Test details | Predicted (LS) mean 1 | Predicted (LS) mean 2 | Predicted (LS) mean diff, | SE of diff, | N1 | N2 | t | DF |
| WT vs. S7KO (+CRE) | 0.8669 | 0.8985 | -0.03159 | 0.005833 | 750 | 650 | 5.416 | 4148 |
| WT vs. S7a Rescue | 0.8669 | 0.8596 | 0.007326 | 0.005621 | 750 | 750 | 1.303 | 4148 |
| WT vs. PM-S7a Rescue | 0.8669 | 0.8674 | -0.0004883 | 0.005625 | 750 | 748 | 0.08681 | 4148 |
| WT vs. Lyso-S7a Rescue | 0.8669 | 0.895 | -0.02815 | 0.005621 | 750 | 750 | 5.009 | 4148 |
| WT vs. SV-S7a Rescue | 0.8669 | 0.9224 | -0.05557 | 0.005532 | 750 | 800 | 10.04 | 4148 |
| S7KO (+CRE) vs. S7a Rescue | 0.8985 | 0.8596 | 0.03892 | 0.005833 | 650 | 750 | 6.672 | 4148 |
| S7KO (+CRE) vs. PM-S7a Rescue | 0.8985 | 0.8674 | 0.0311 | 0.005837 | 650 | 748 | 5.329 | 4148 |
| S7KO (+CRE) vs. Lyso-S7a Rescue | 0.8985 | 0.895 | 0.003441 | 0.005833 | 650 | 750 | 0.5899 | 4148 |
| S7KO (+CRE) vs. SV-S7a Rescue | 0.8985 | 0.9224 | -0.02397 | 0.005748 | 650 | 800 | 4.171 | 4148 |
| S7a Rescue vs. PM-S7a Rescue | 0.8596 | 0.8674 | -0.007814 | 0.005625 | 750 | 748 | 1.389 | 4148 |
| S7a Rescue vs. Lyso-S7a Rescue | 0.8596 | 0.895 | -0.03548 | 0.005621 | 750 | 750 | 6.312 | 4148 |
| S7a Rescue vs. SV-S7a Rescue | 0.8596 | 0.9224 | -0.06289 | 0.005532 | 750 | 800 | 11.37 | 4148 |
| PM-S7a Rescue vs. Lyso-S7a Rescue | 0.8674 | 0.895 | -0.02766 | 0.005625 | 748 | 750 | 4.918 | 4148 |
| PM-S7a Rescue vs. SV-S7a Rescue | 0.8674 | 0.9224 | -0.05508 | 0.005536 | 748 | 800 | 9.948 | 4148 |
| Lyso-S7a Rescue vs. SV-S7a Rescue | 0.895 | 0.9224 | -0.02741 | 0.005532 | 750 | 800 | 4.955 | 4148 |
