# Supplementary material for: Synaptotagmin 7 is targeted to the axonal plasma membrane through γ-secretase processing to promote synaptic vesicle docking in mouse hippocampal neurons
Source: eLife. 2021 Sep 20;10:e67261. doi: 10.7554/eLife.67261 (PMC8452306; doi:10.7554/eLife.67261)
Supplement: Source data 1. [file elife-67261-data1.zip › Source data files/uncropped blots and gels with labels - 04-02-2021-RA-eLife-67261.pdf]

Figure 4 - Figure Supplement 2a

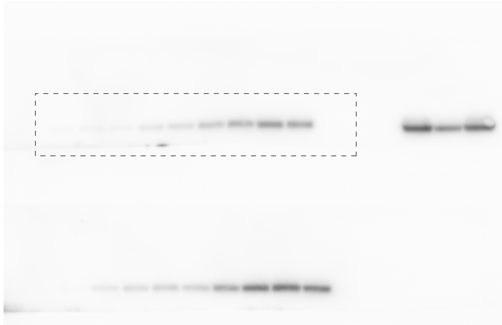

Uncropped anti SYT1 blot

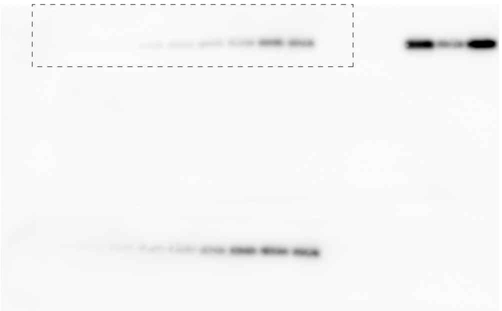

Uncropped anti SYP blot

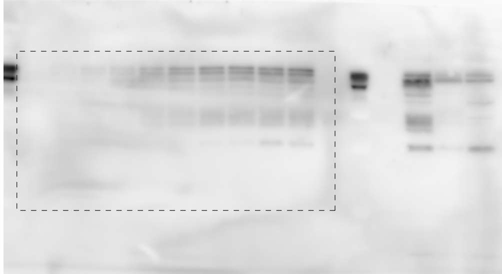

Uncropped anti SYT7 blot

Figure 4 - Figure Supplement 3a

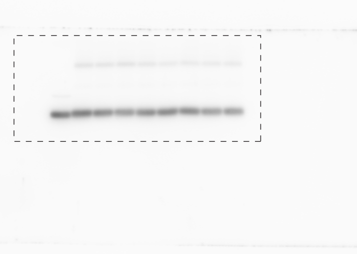

Uncropped anti SYP blot

Figure 5a

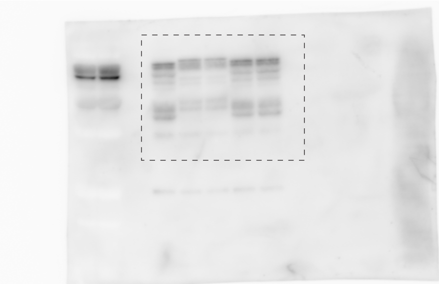

Uncropped anti SYT7 blot

Figure 5b

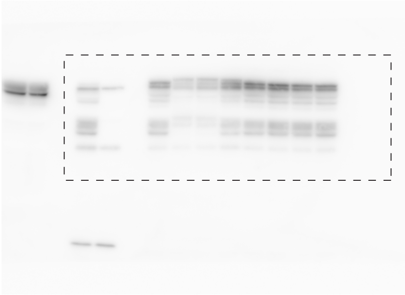

Uncropped anti SYT7 blot

Figure 5 - figure supplement 1a

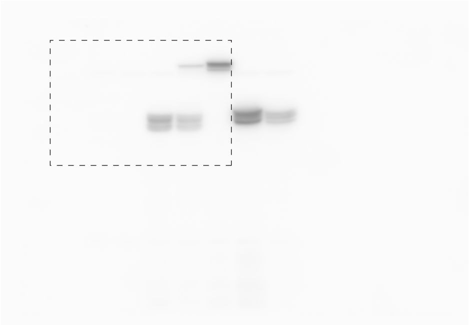

Uncropped anti SYT7 blot

Figure 5 - figure supplement 1b

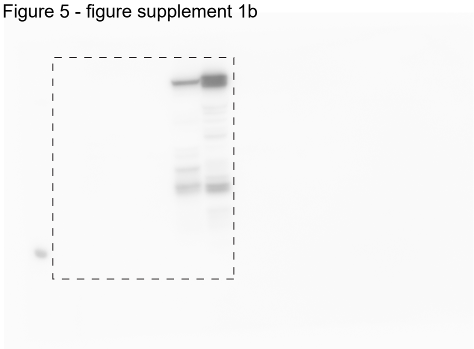

Uncropped anti SYT7 blot

Figure 5 - figure supplement 1c-d

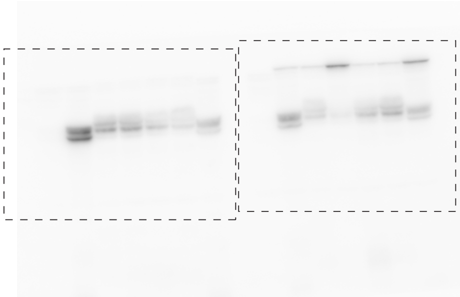

Uncropped anti SYT7 blot

Figure 6e

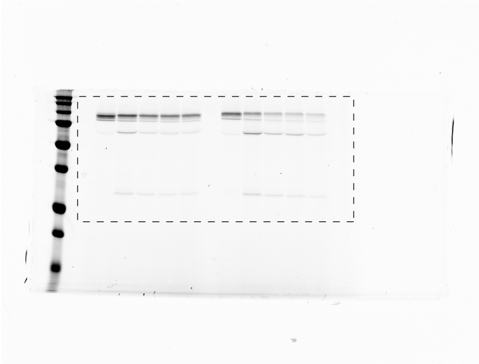

Uncropped in-gel fluorescence

Figure 6 - figure supplement 1d-e

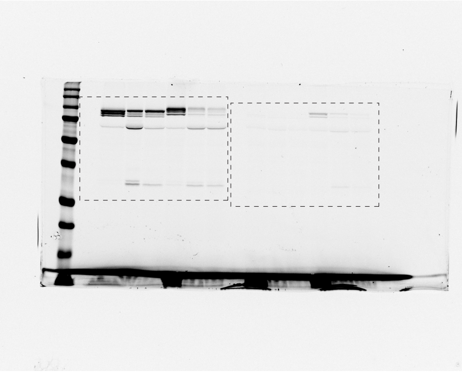

Uncropped in-gel fluorescence

Figure 7 - figure supplement 1g

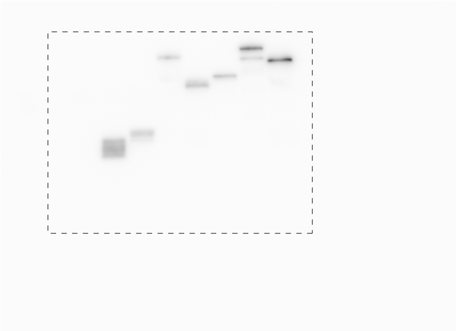

Uncropped anti SYT7 blot
