# Supplementary figures and images for: Synaptotagmin 7 is targeted to the axonal plasma membrane through γ-secretase processing to promote synaptic vesicle docking in mouse hippocampal neurons
Source: eLife. 2021 Sep 20;10:e67261. doi: 10.7554/eLife.67261 (PMC8452306; doi:10.7554/eLife.67261)

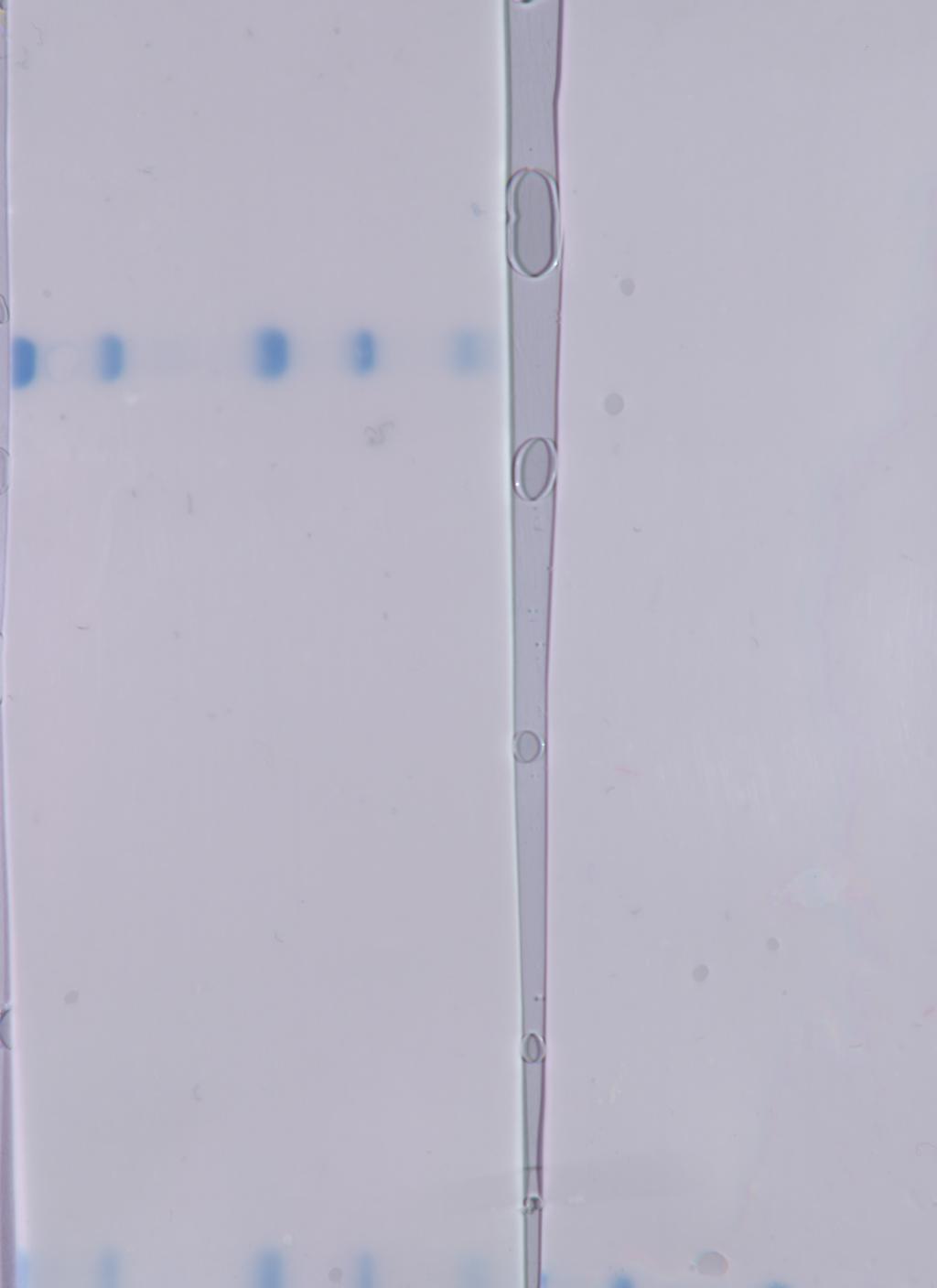

Supplement: Source data 1. [file elife-67261-data1.zip › Source data files/Raw blot images/Figure 4 - Figure Supplement 2/Figure 4 - Figure Supplement 2a anti-SYP.jpg]

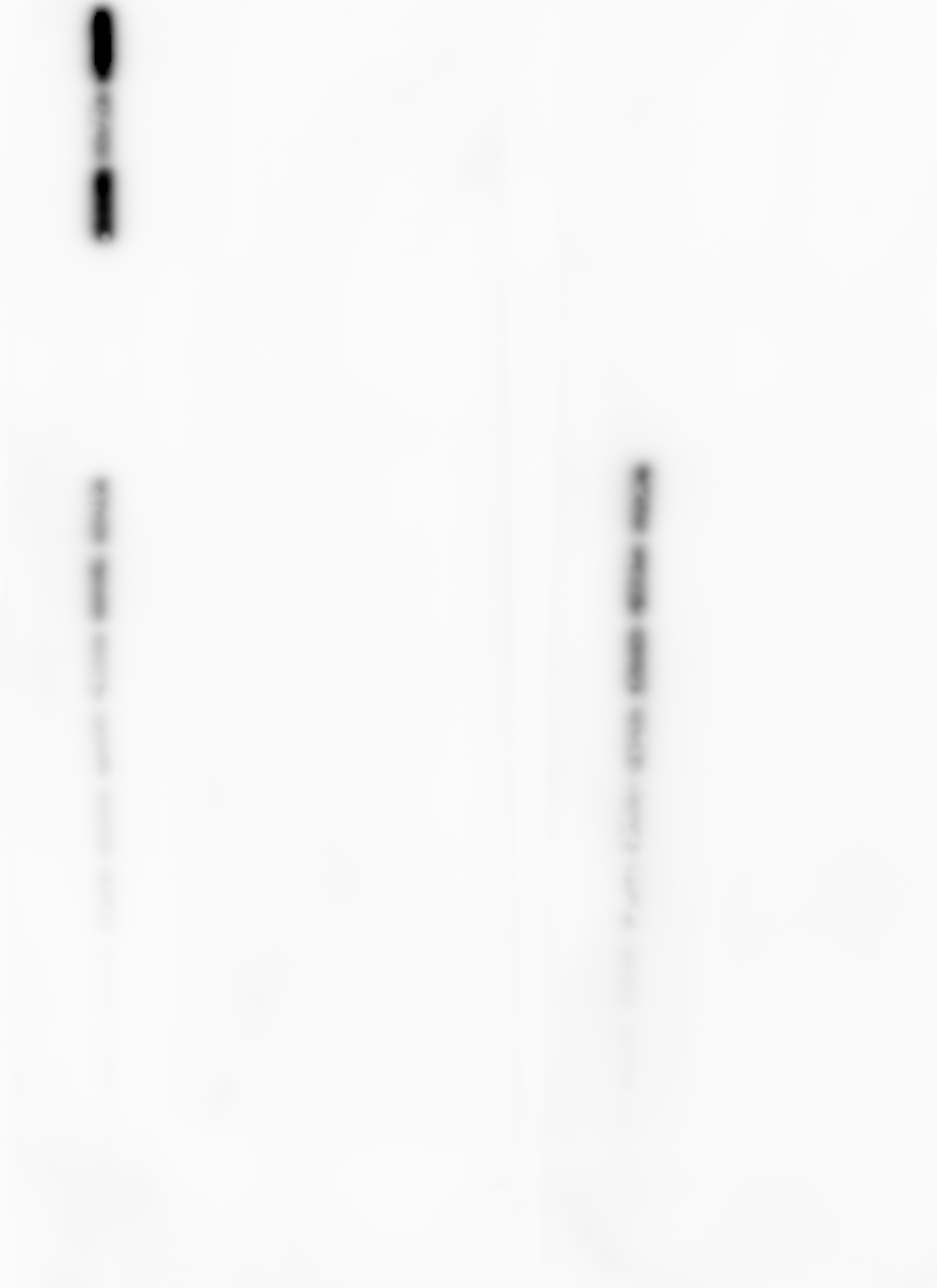

Supplement: Source data 1. [file elife-67261-data1.zip › Source data files/Raw blot images/Figure 4 - Figure Supplement 2/Figure 4 - Figure Supplement 2a anti-SYP.tif]

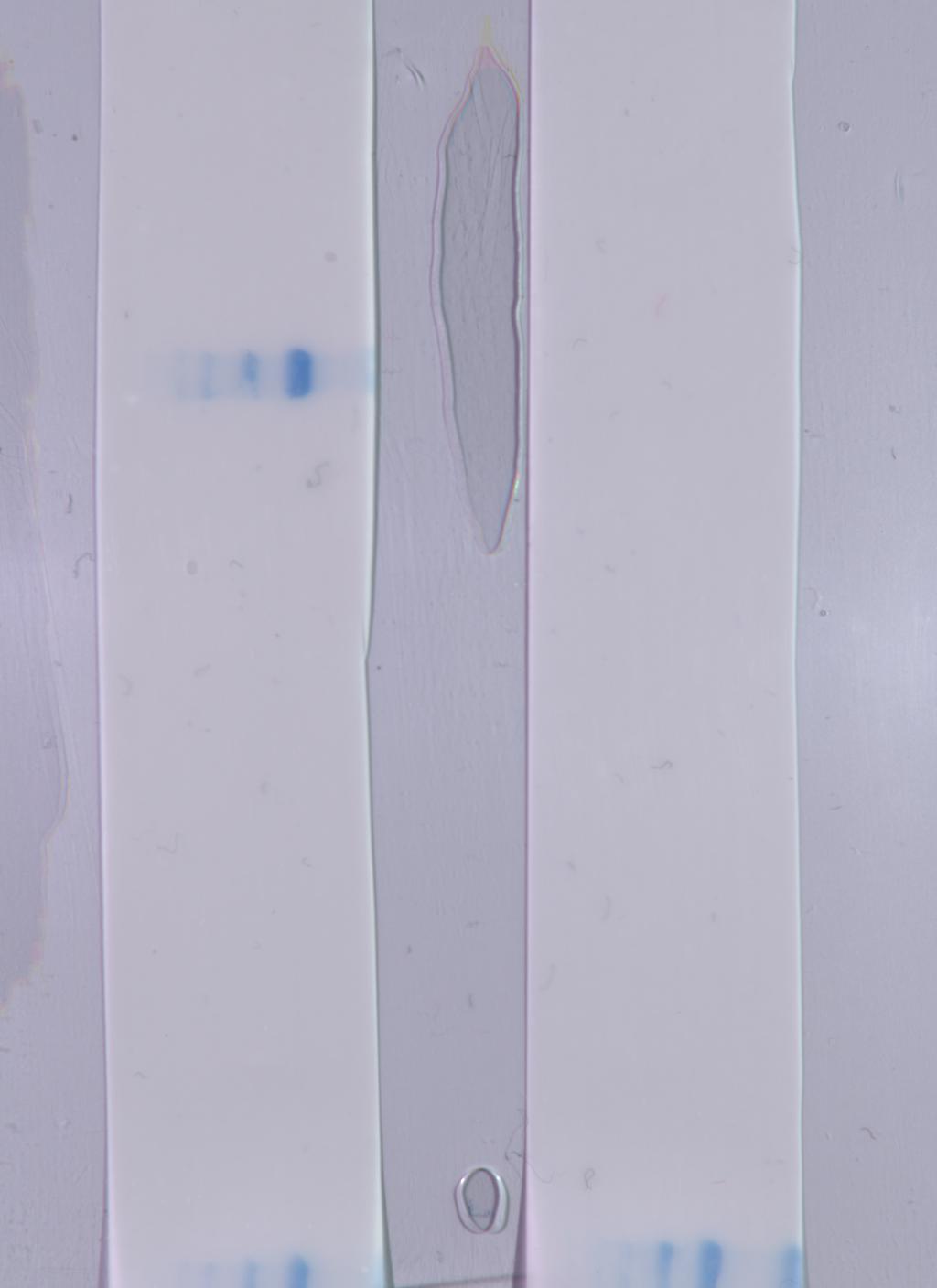

Supplement: Source data 1. [file elife-67261-data1.zip › Source data files/Raw blot images/Figure 4 - Figure Supplement 2/Figure 4 - Figure Supplement 2a anti-SYT1 Ladder.jpg]

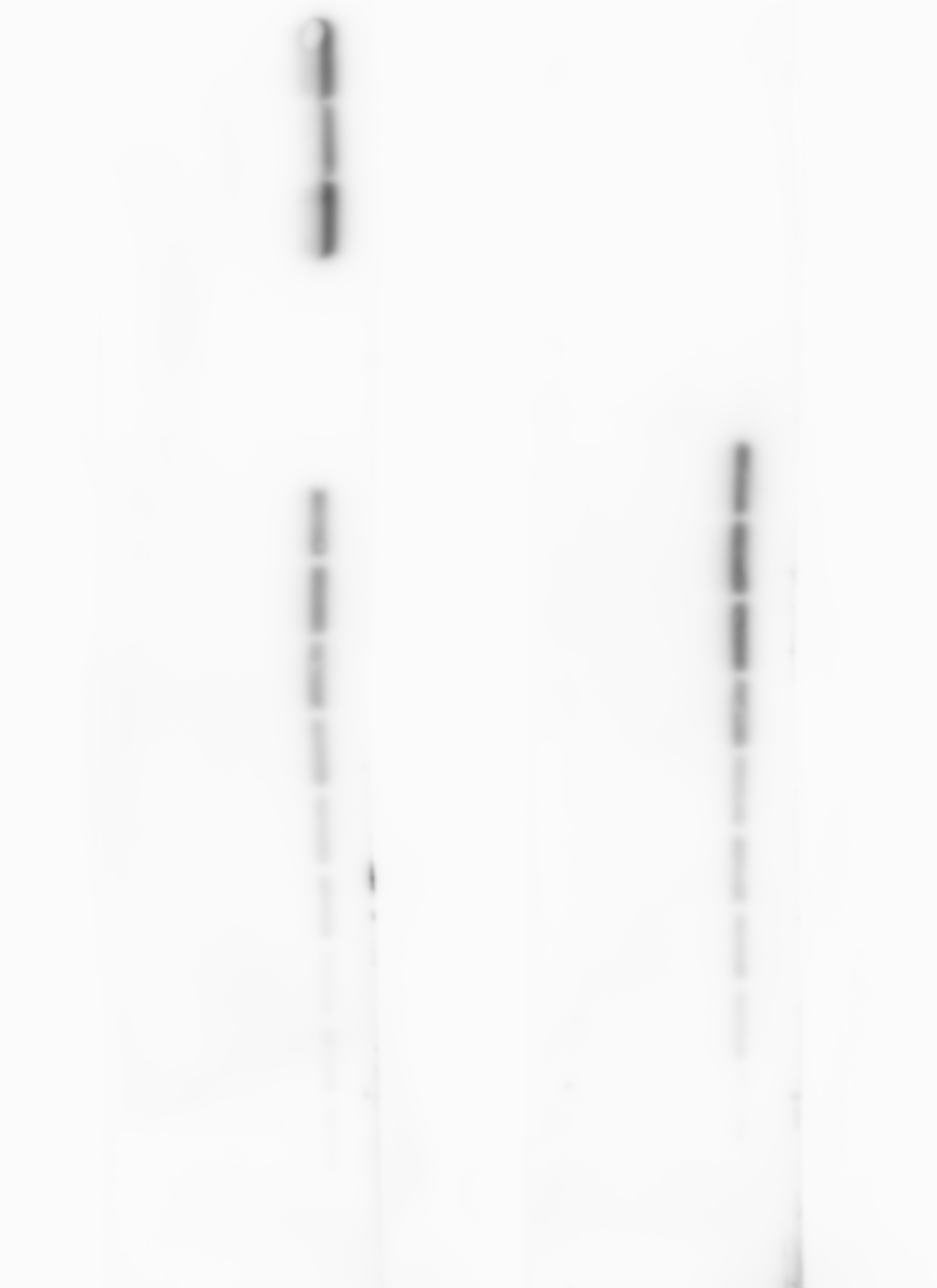

Supplement: Source data 1. [file elife-67261-data1.zip › Source data files/Raw blot images/Figure 4 - Figure Supplement 2/Figure 4 - Figure Supplement 2a anti-SYT1.tif]

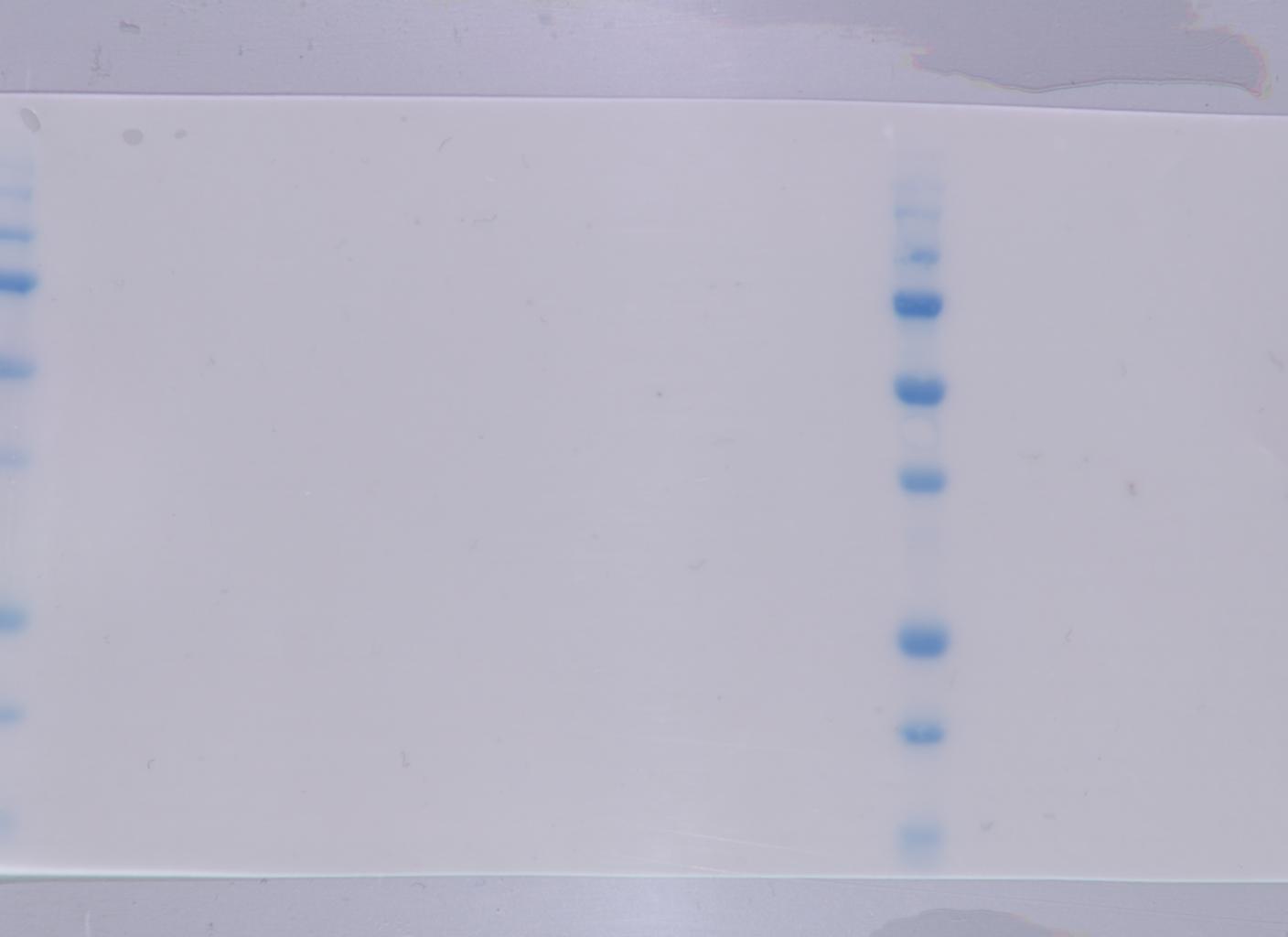

Supplement: Source data 1. [file elife-67261-data1.zip › Source data files/Raw blot images/Figure 4 - Figure Supplement 2/Figure 4 - Figure Supplement 2a anti-SYT7 Ladder.jpg]

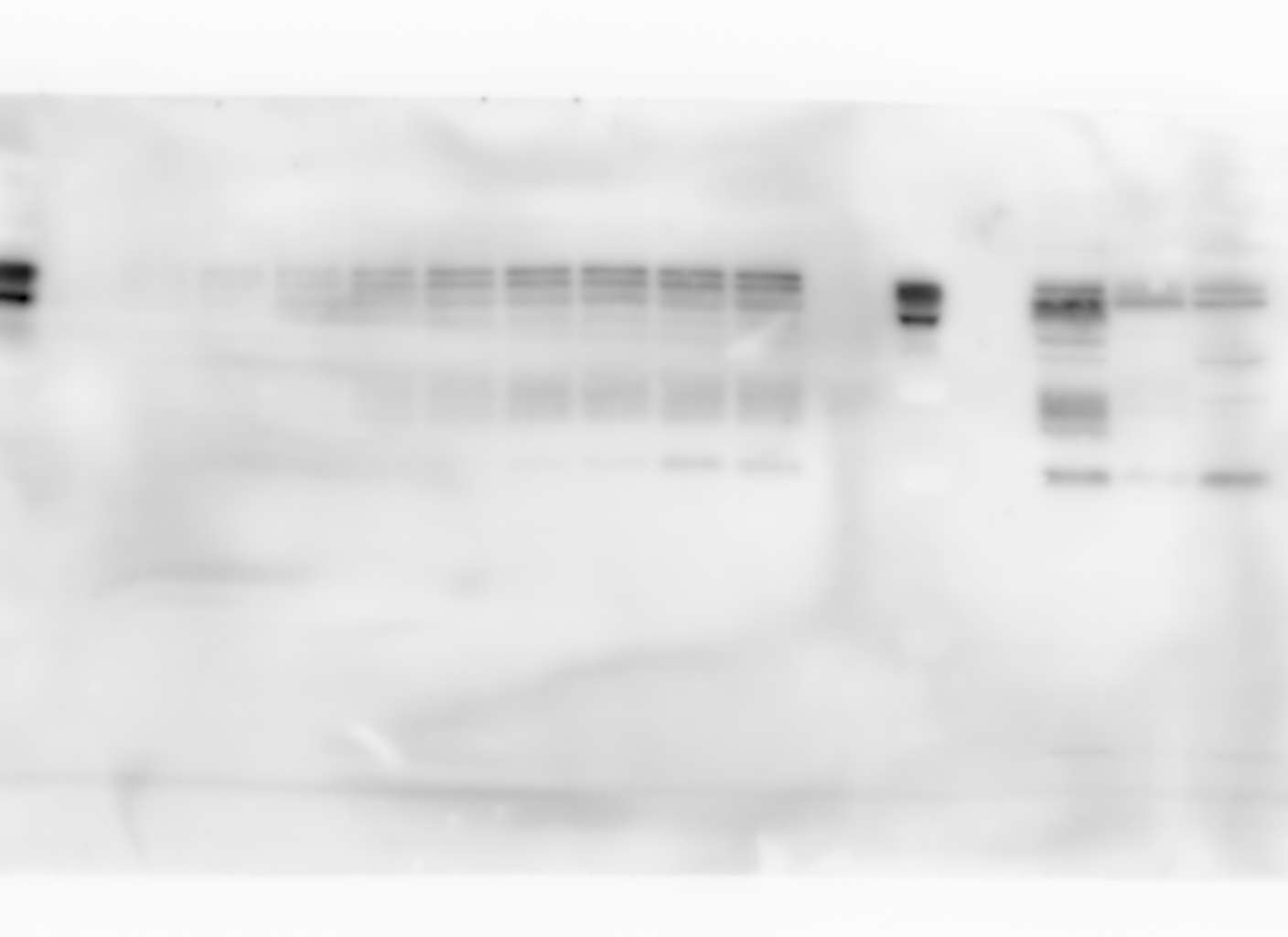

Supplement: Source data 1. [file elife-67261-data1.zip › Source data files/Raw blot images/Figure 4 - Figure Supplement 2/Figure 4 - Figure Supplement 2a anti-SYT7.tif]

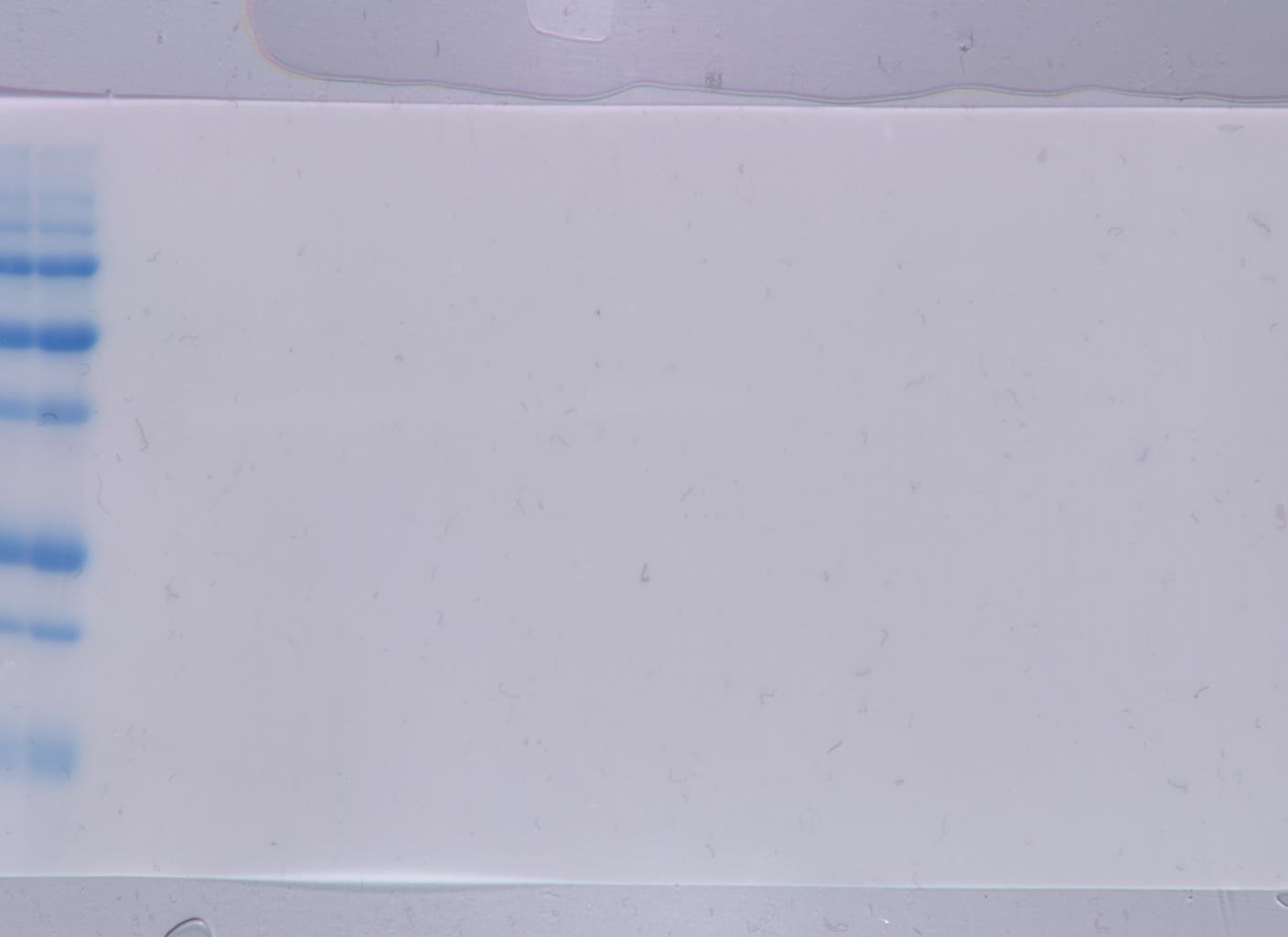

Supplement: Source data 1. [file elife-67261-data1.zip › Source data files/Raw blot images/Figure 4 - Figure Supplement 3/Figure 4 - Figure Supplement 3a anti-SYP.jpg]

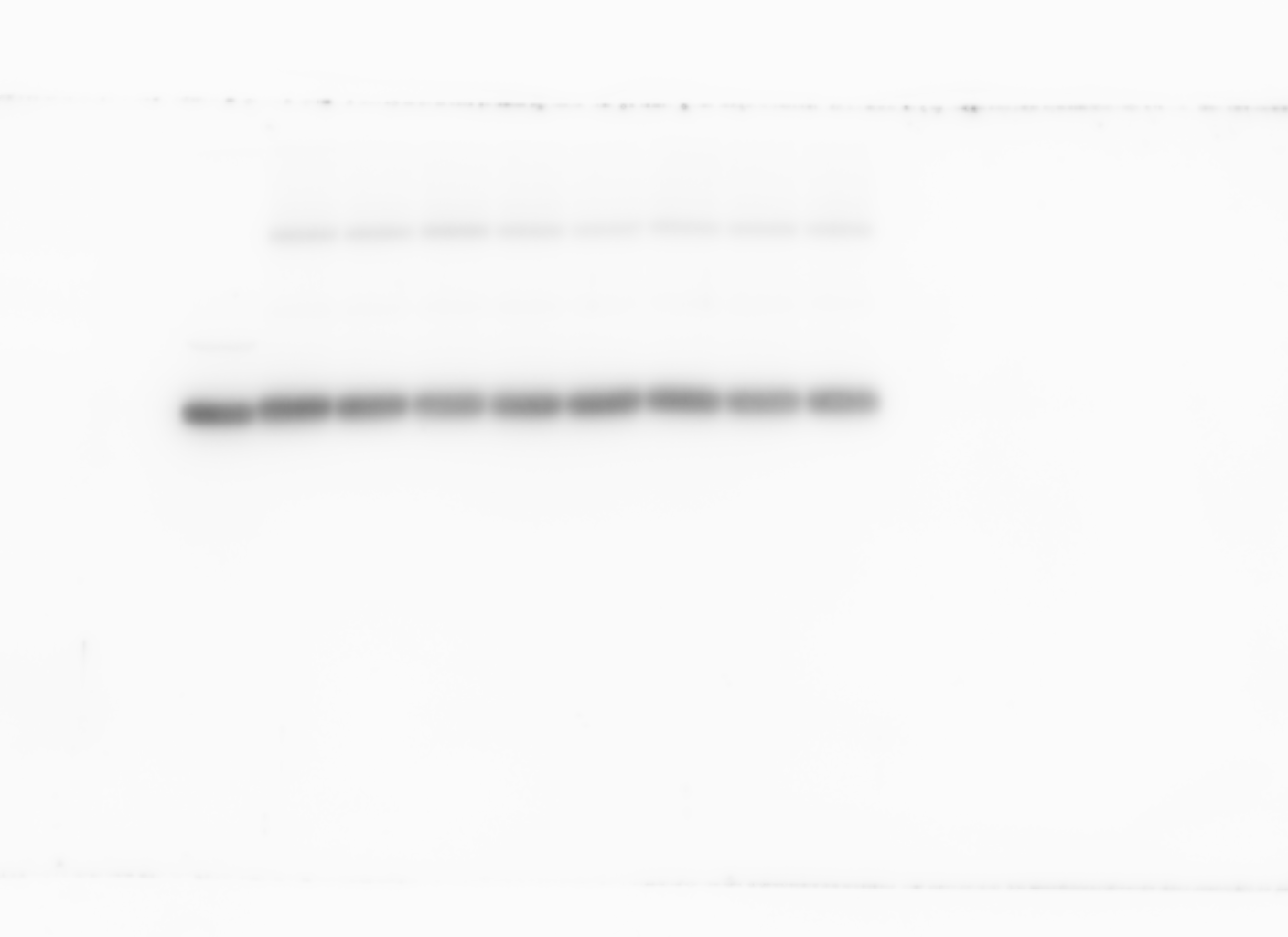

Supplement: Source data 1. [file elife-67261-data1.zip › Source data files/Raw blot images/Figure 4 - Figure Supplement 3/Figure 4 - Figure Supplement 3a anti-SYP.tif]

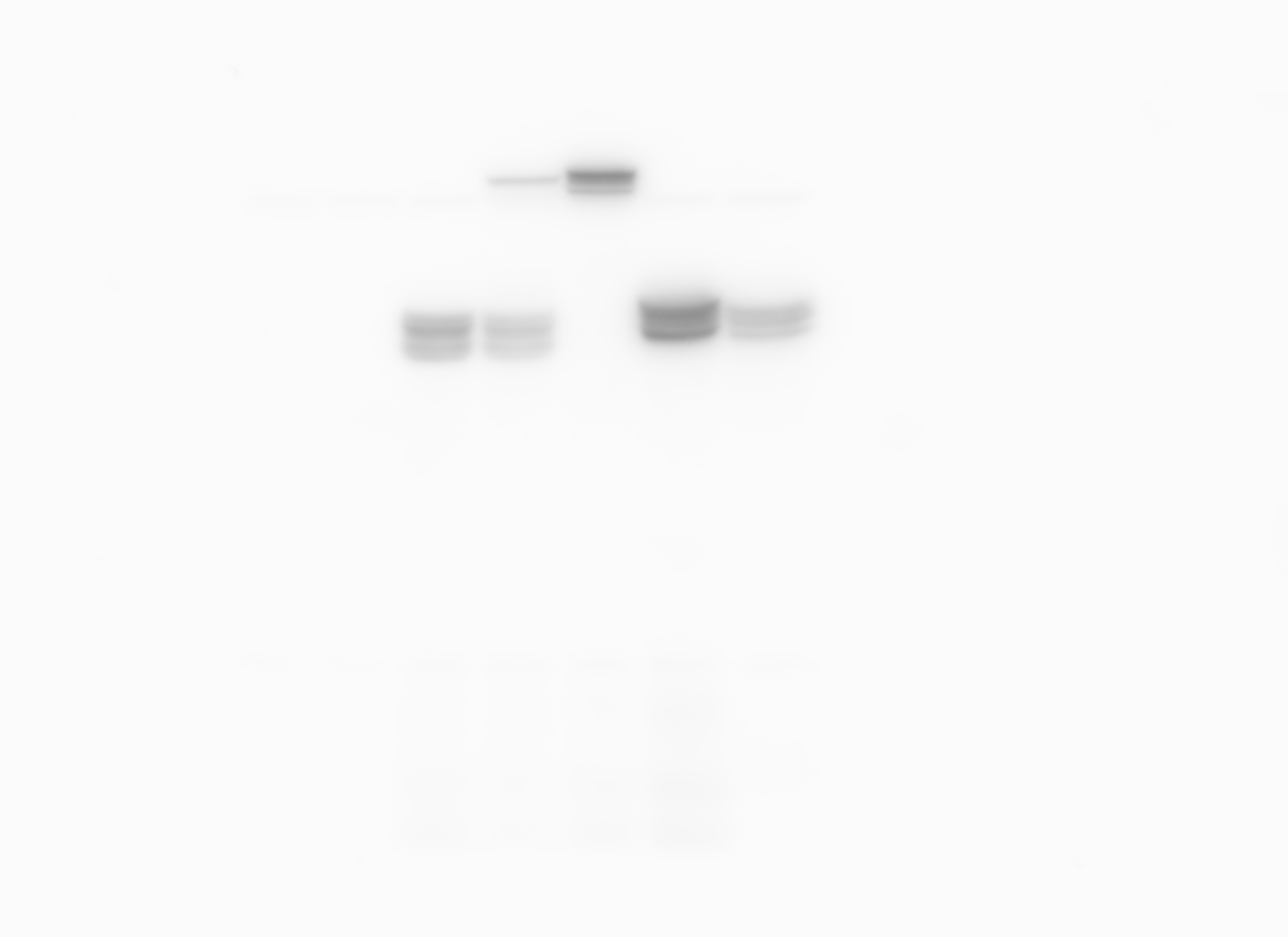

Supplement: Source data 1. [file elife-67261-data1.zip › Source data files/Raw blot images/Figure 5 - figure supplement 1/Figure 5 - figure supplement 1a anti-SYT7.tif]

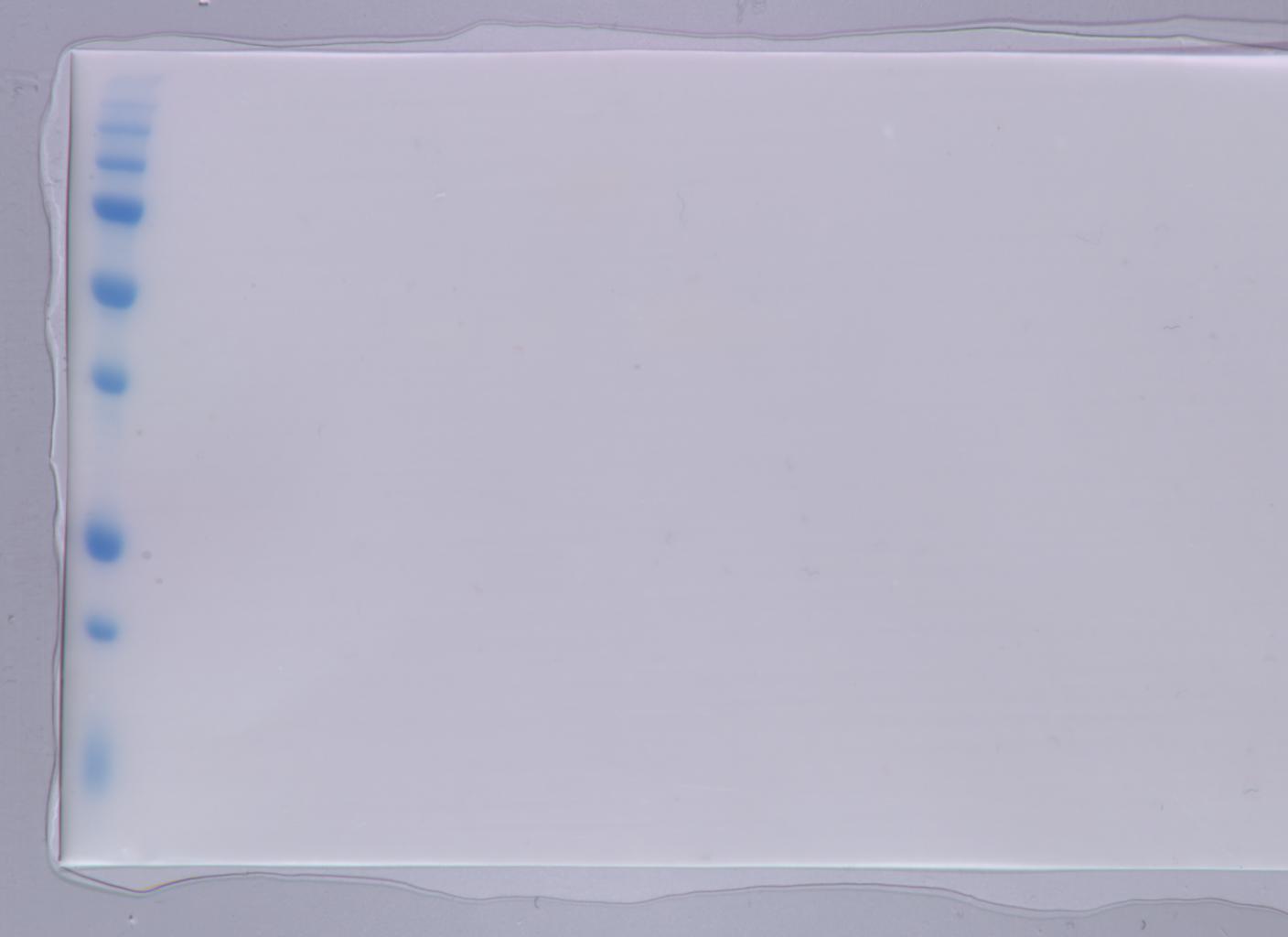

Supplement: Source data 1. [file elife-67261-data1.zip › Source data files/Raw blot images/Figure 5 - figure supplement 1/Figure 5 - figure supplement 1a Ladder anti-SYT7.jpg]

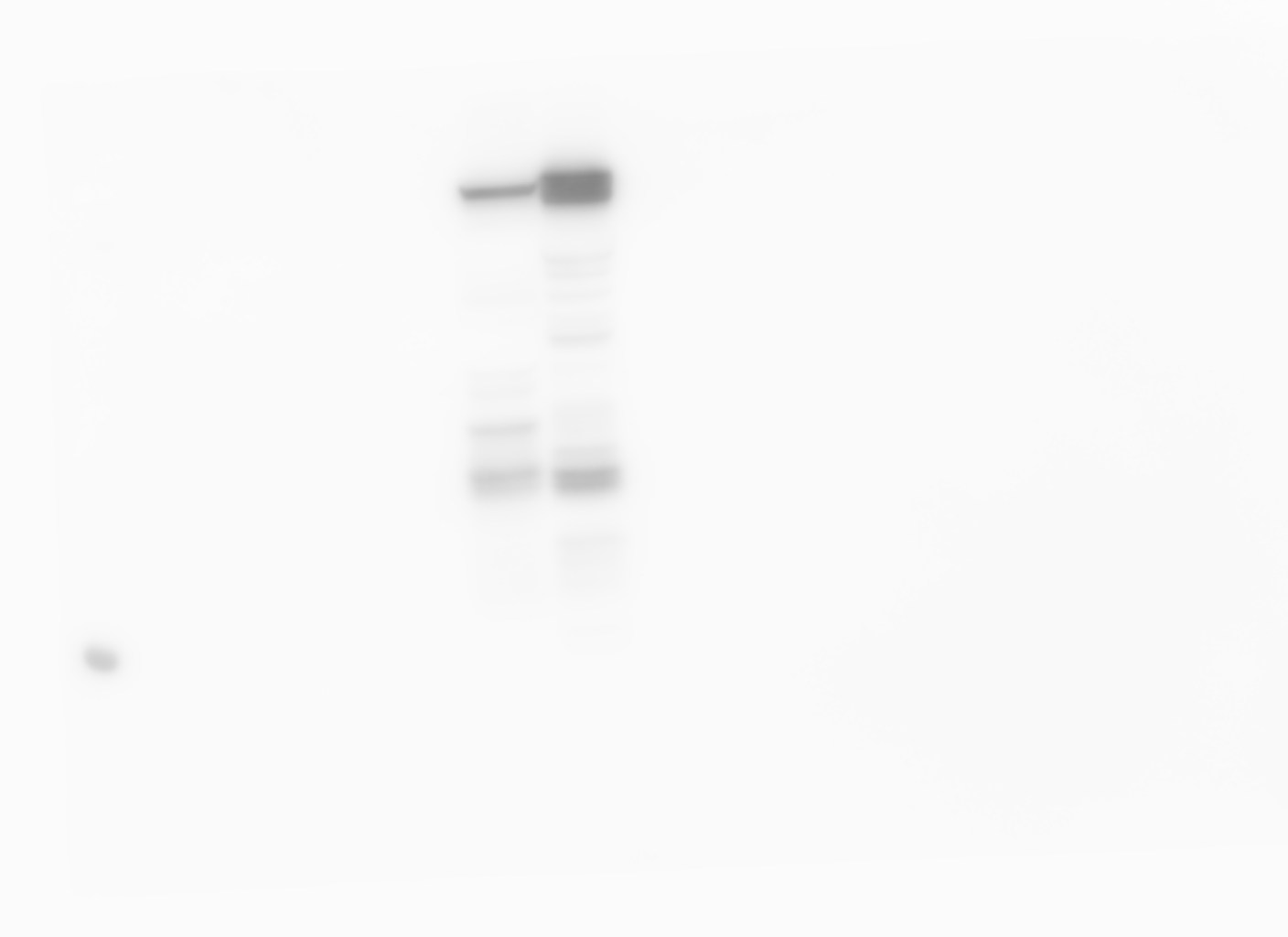

Supplement: Source data 1. [file elife-67261-data1.zip › Source data files/Raw blot images/Figure 5 - figure supplement 1/Figure 5 - figure supplement 1b anti-Halotag.tif]

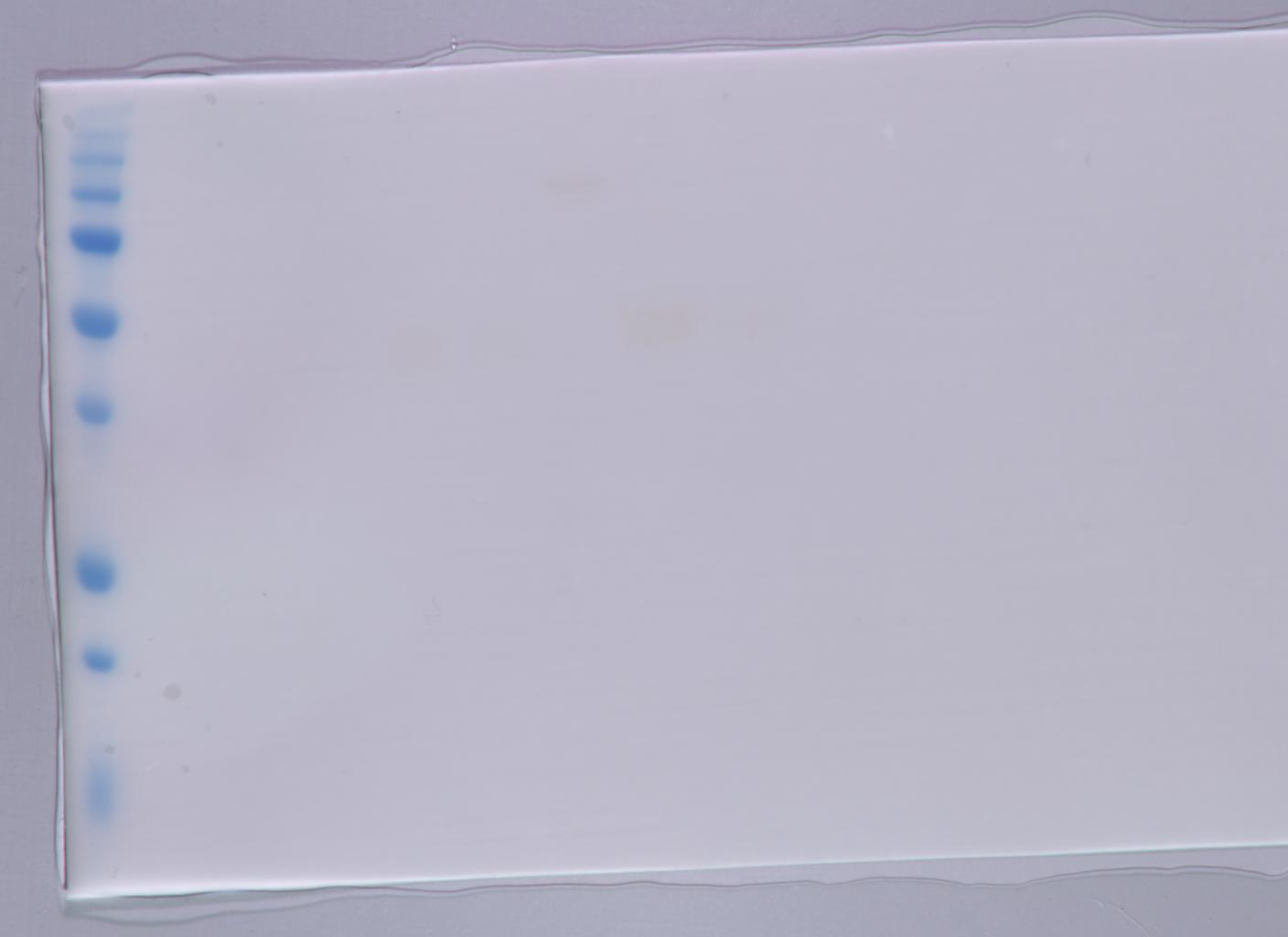

Supplement: Source data 1. [file elife-67261-data1.zip › Source data files/Raw blot images/Figure 5 - figure supplement 1/Figure 5 - figure supplement 1b Ladder for anti-HaloTag.jpg]

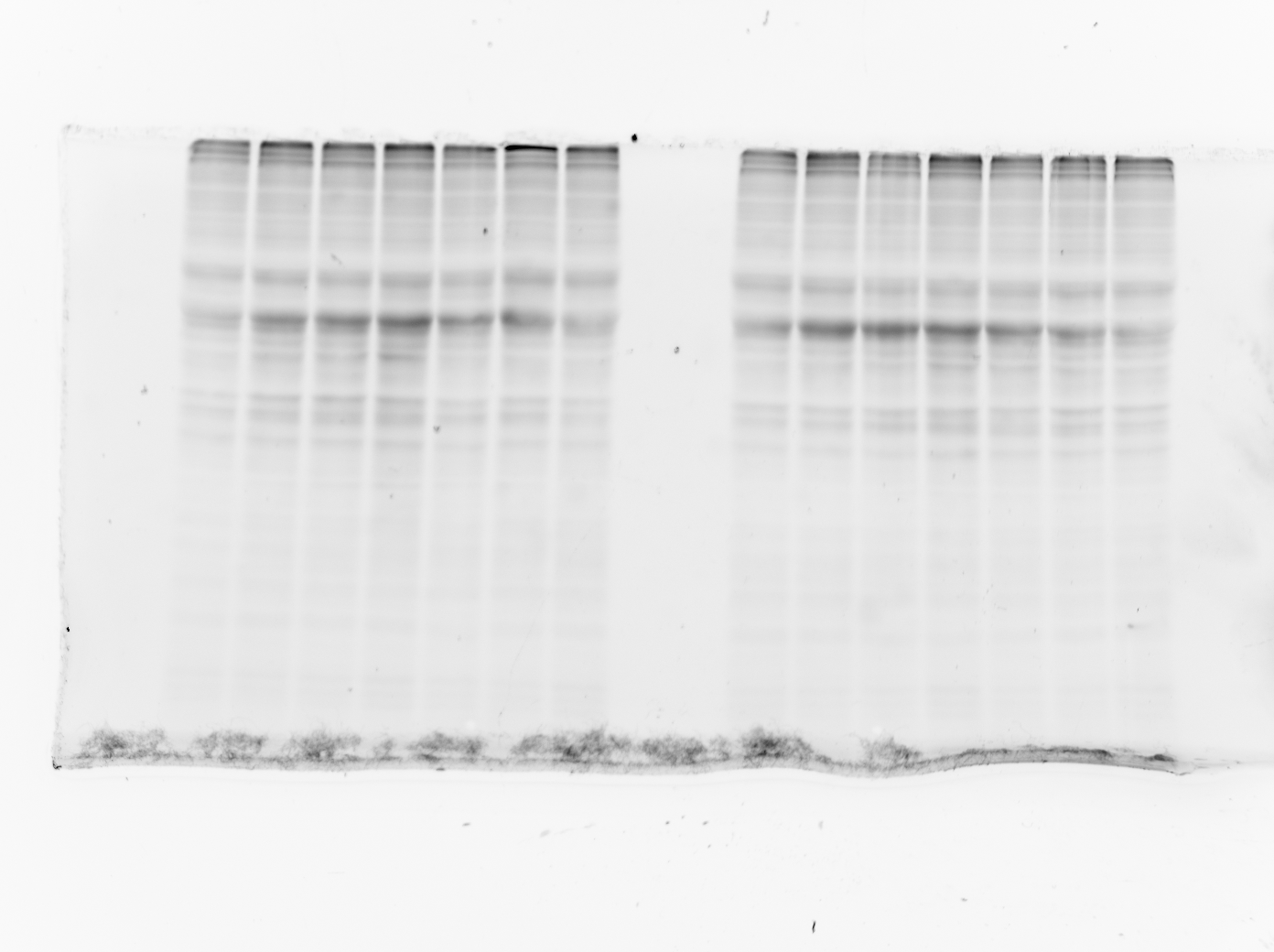

Supplement: Source data 1. [file elife-67261-data1.zip › Source data files/Raw blot images/Figure 5 - figure supplement 1/Figure 5 - figure supplement 1c-d anti-SYT7 TCE load ctrl.tif]

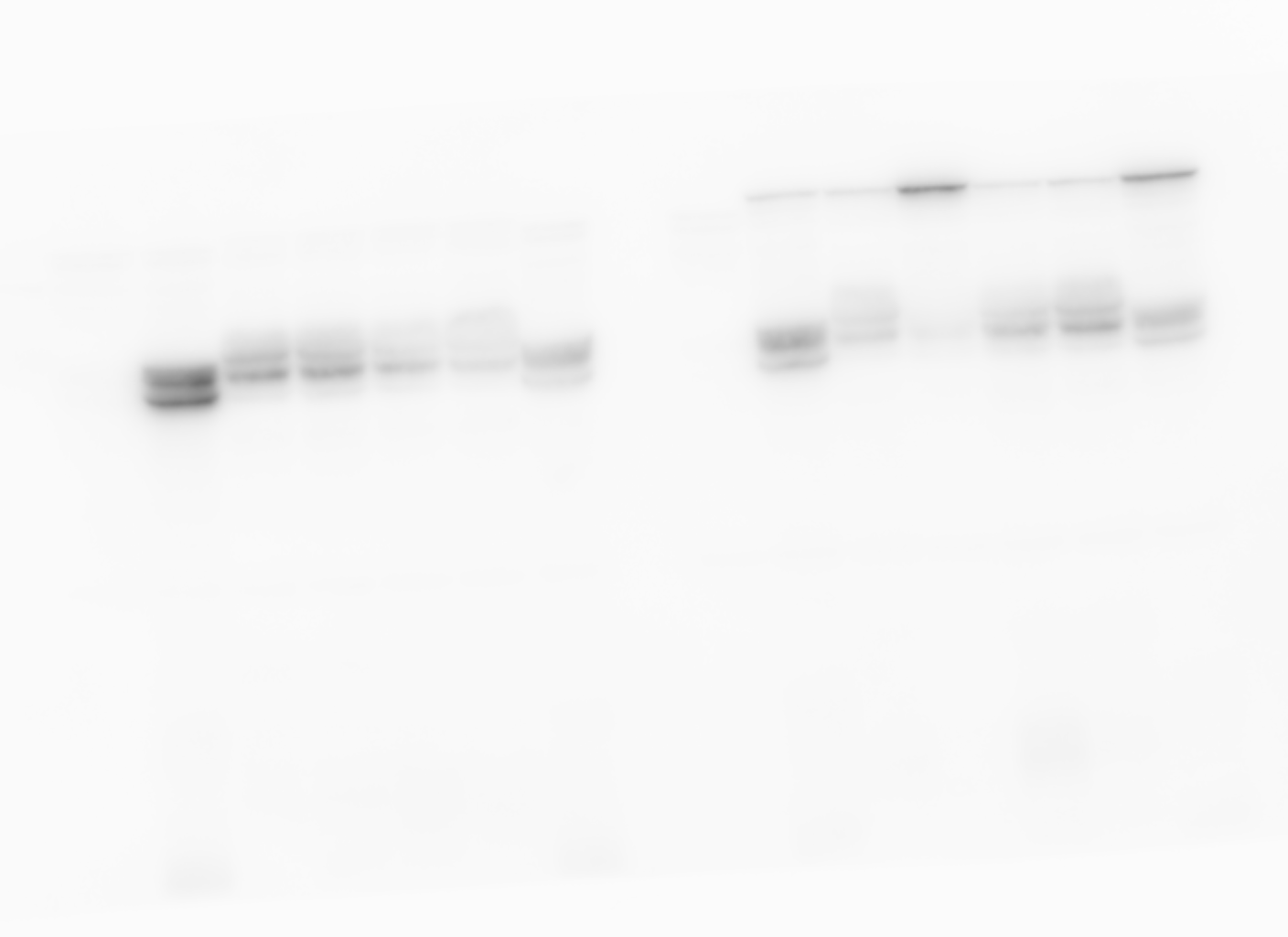

Supplement: Source data 1. [file elife-67261-data1.zip › Source data files/Raw blot images/Figure 5 - figure supplement 1/Figure 5 - figure supplement 1c-d anti-SYT7.tif]

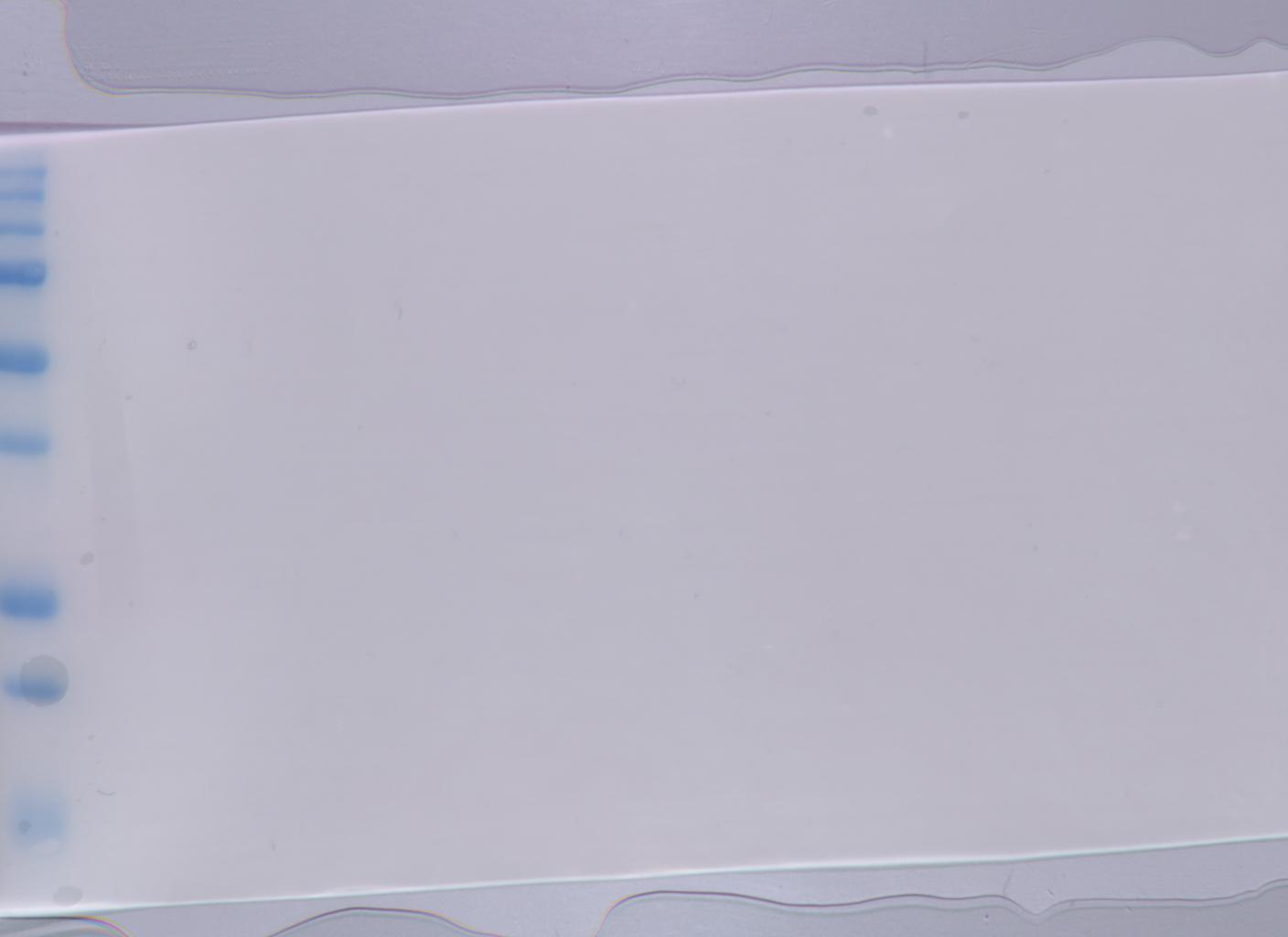

Supplement: Source data 1. [file elife-67261-data1.zip › Source data files/Raw blot images/Figure 5 - figure supplement 1/Figure 5 - figure supplement 1c-d Ladder anti-SYT7.jpg]

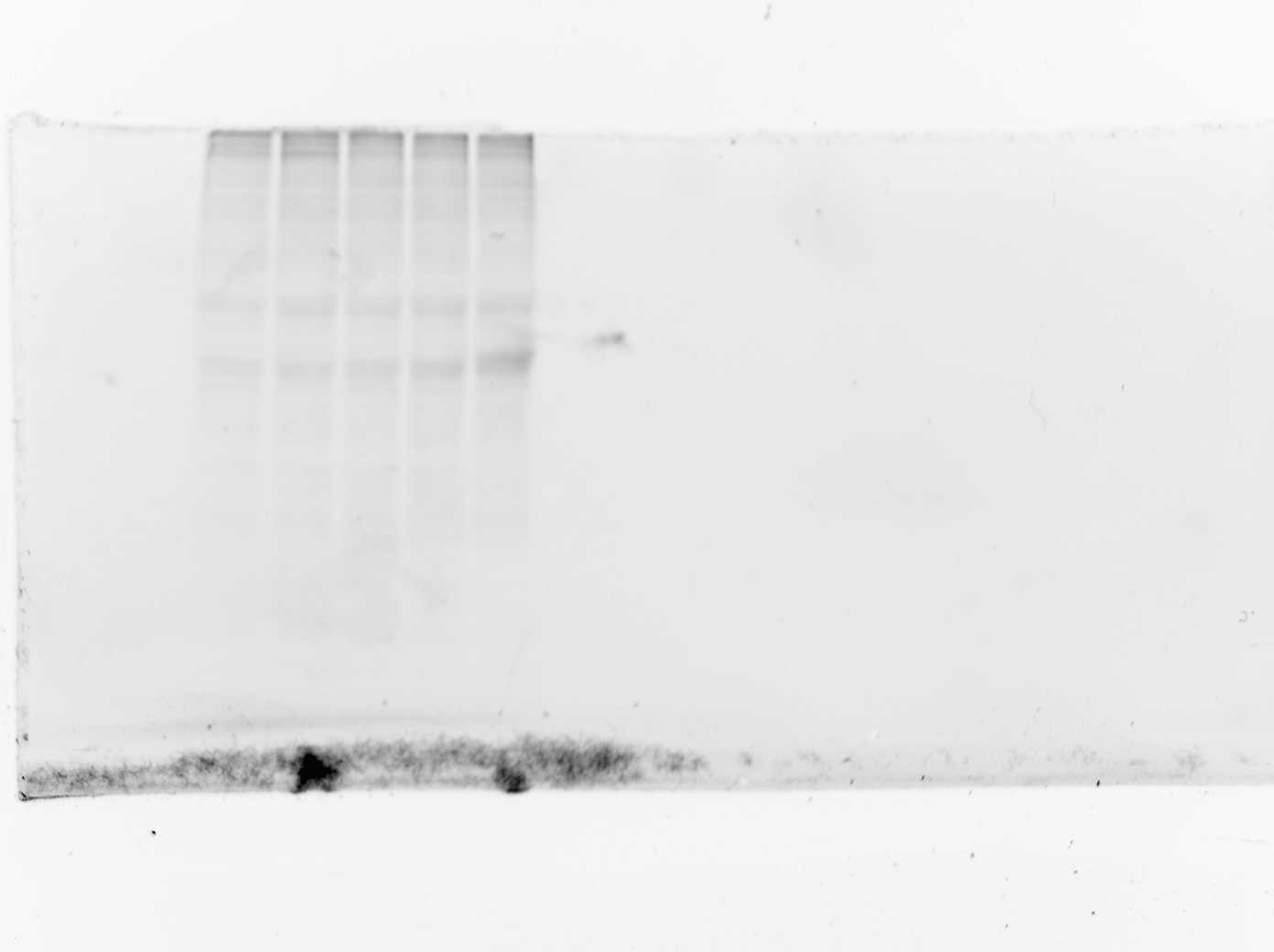

Supplement: Source data 1. [file elife-67261-data1.zip › Source data files/Raw blot images/Figure 5/Figure 5a anti-SYT7 TCE load ctrl.tif]

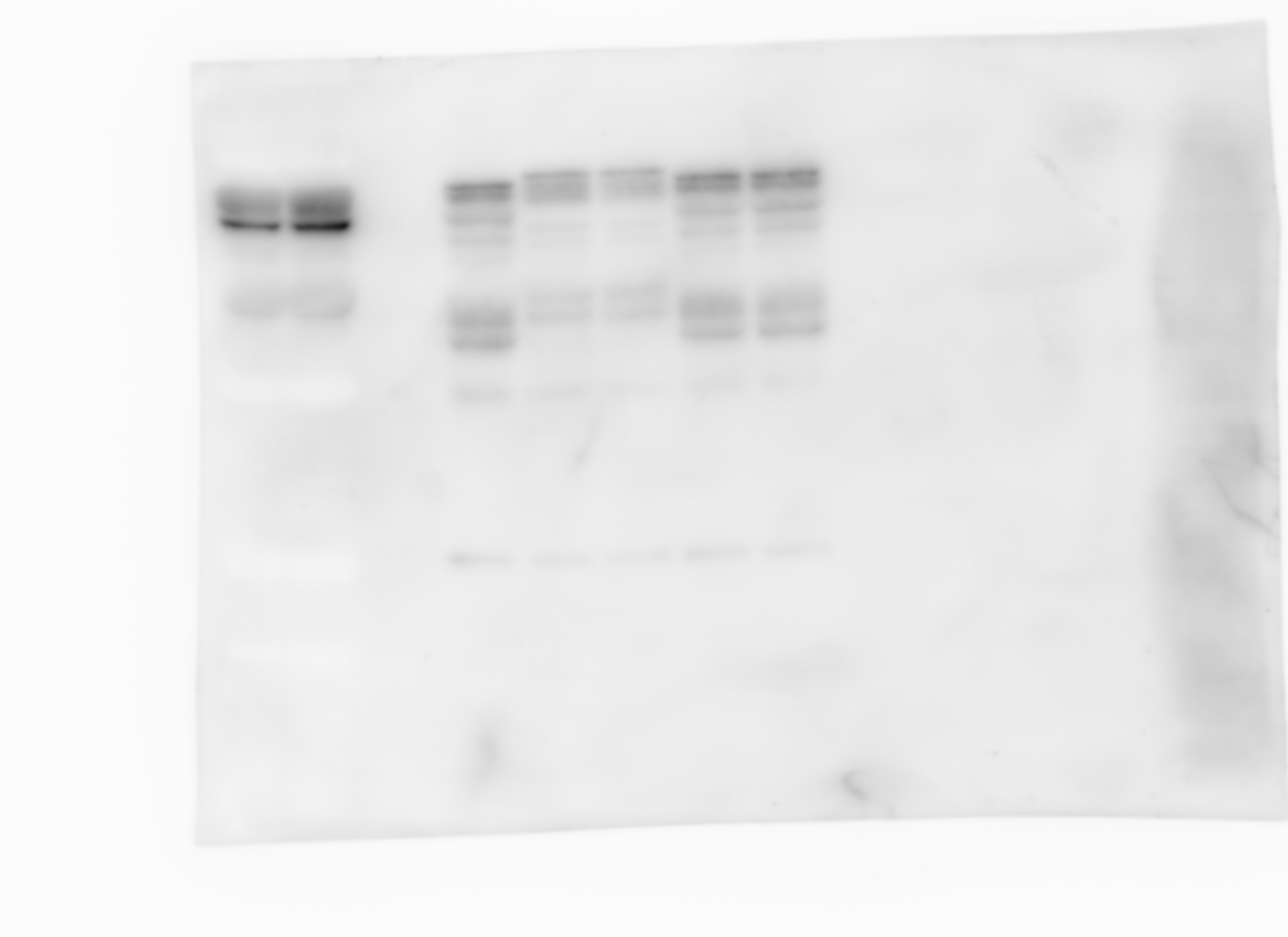

Supplement: Source data 1. [file elife-67261-data1.zip › Source data files/Raw blot images/Figure 5/Figure 5a anti-SYT7.tif]

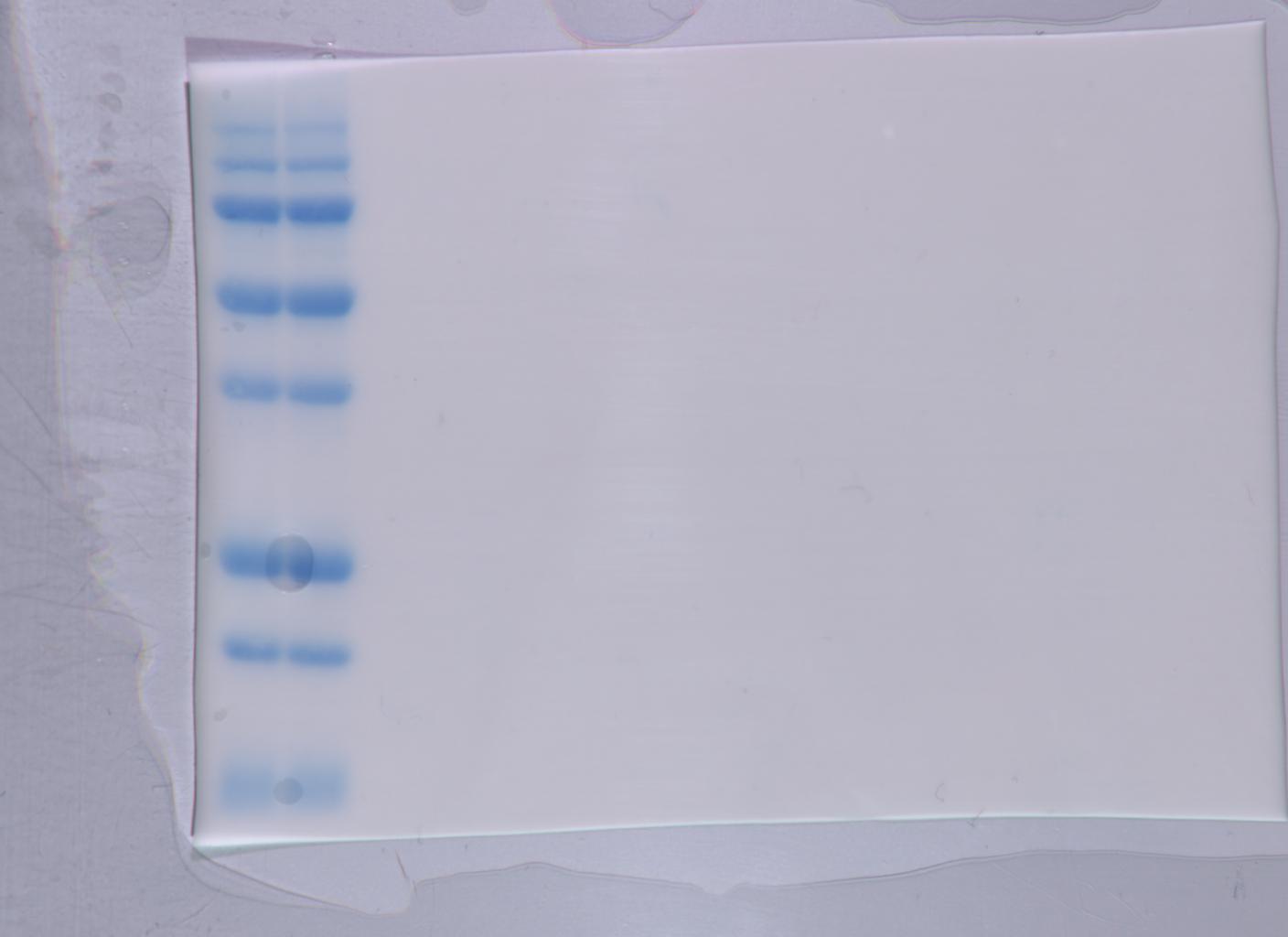

Supplement: Source data 1. [file elife-67261-data1.zip › Source data files/Raw blot images/Figure 5/Figure 5a Ladder anti-SYT7.jpg]

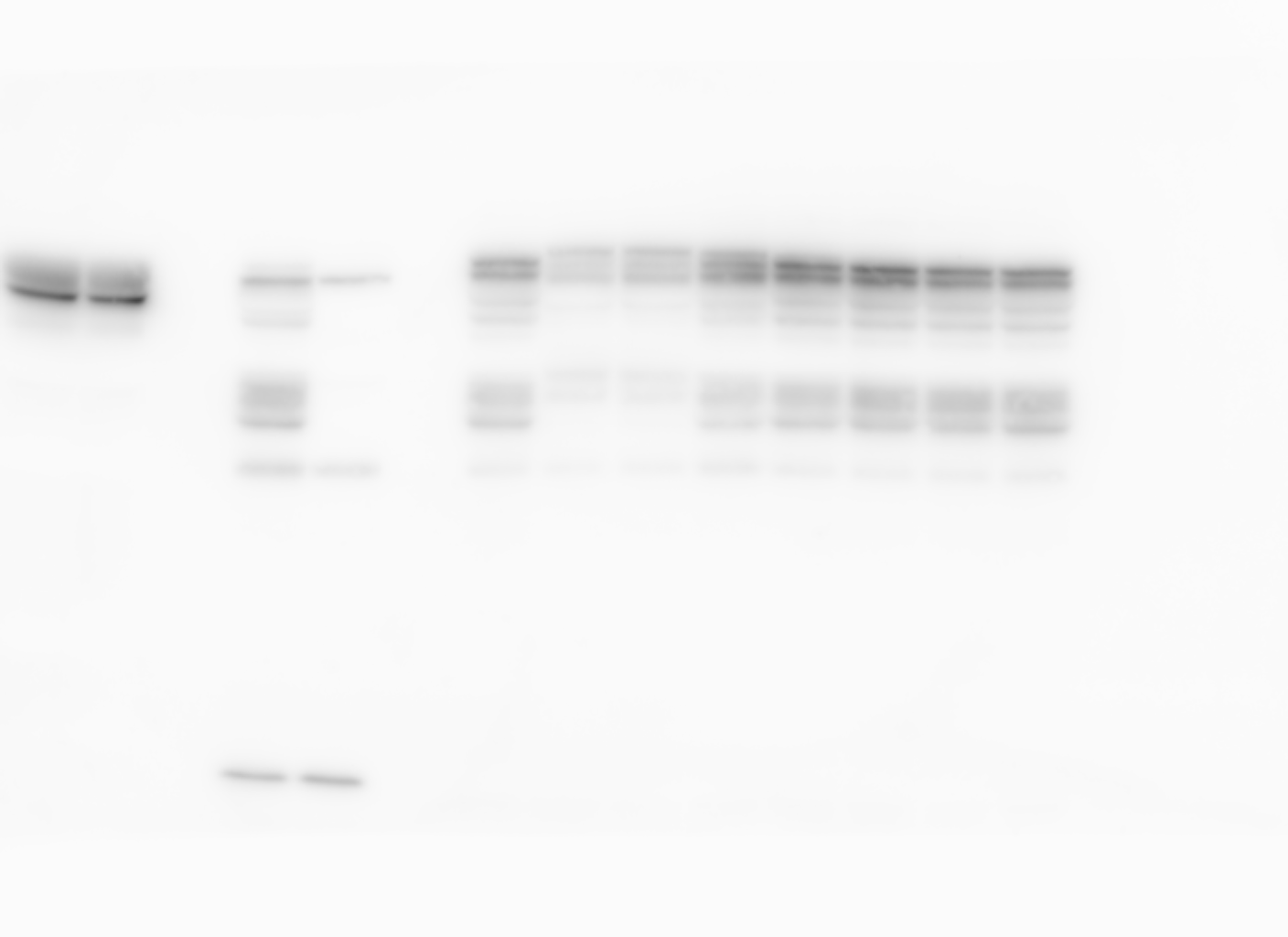

Supplement: Source data 1. [file elife-67261-data1.zip › Source data files/Raw blot images/Figure 5/Figure 5b anti-SYT7.tif]

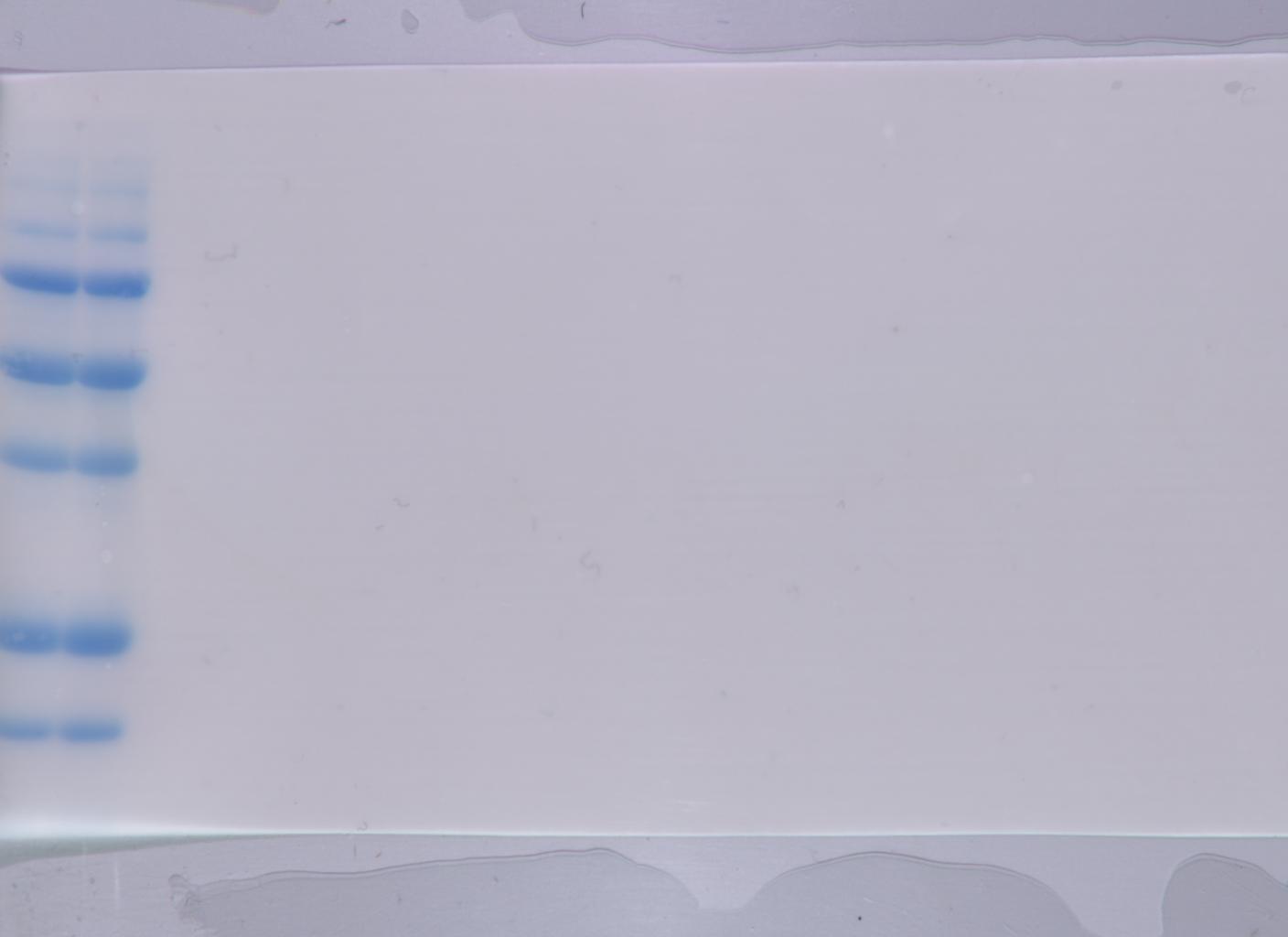

Supplement: Source data 1. [file elife-67261-data1.zip › Source data files/Raw blot images/Figure 5/Figure 5b Ladder anti-SYT7.jpg]

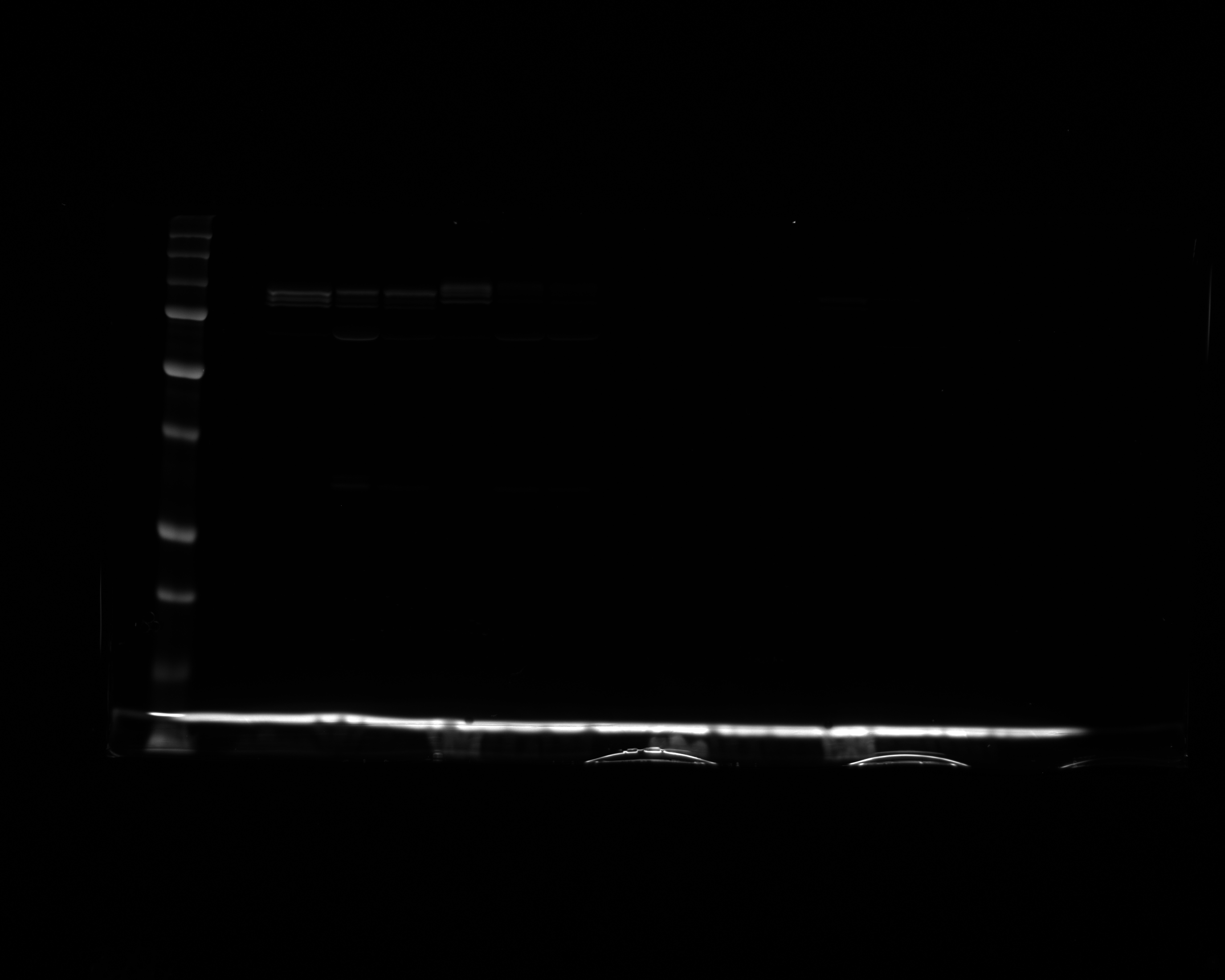

Supplement: Source data 1. [file elife-67261-data1.zip › Source data files/Raw blot images/Figure 6 - Figure Supplement 1/Figure 6 - Figure Supplement 1d-e in-gel fluorescence far red.tif]

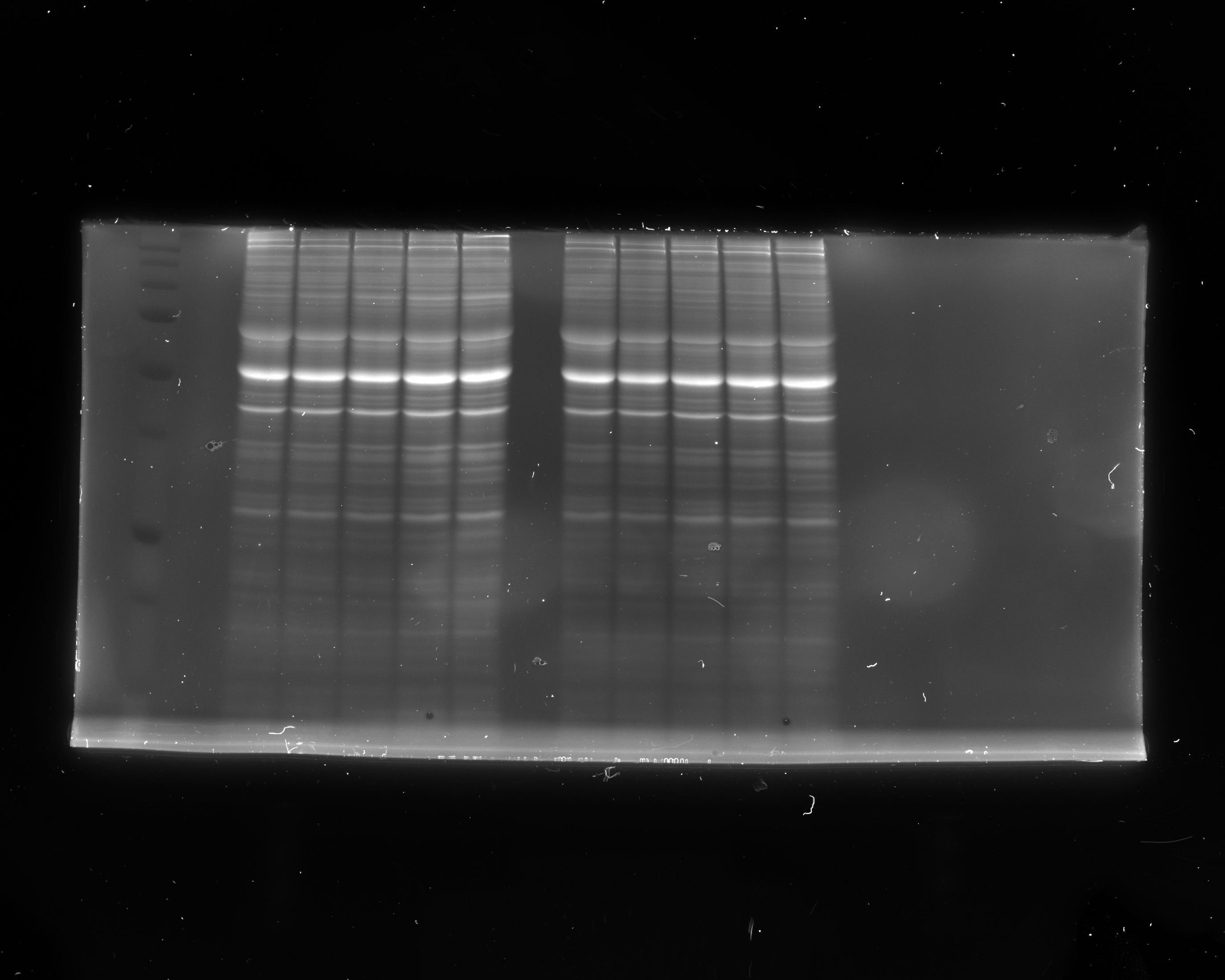

Supplement: Source data 1. [file elife-67261-data1.zip › Source data files/Raw blot images/Figure 6/Figure 6e in-gel far red fluorescence TCE load ctrl.tif]

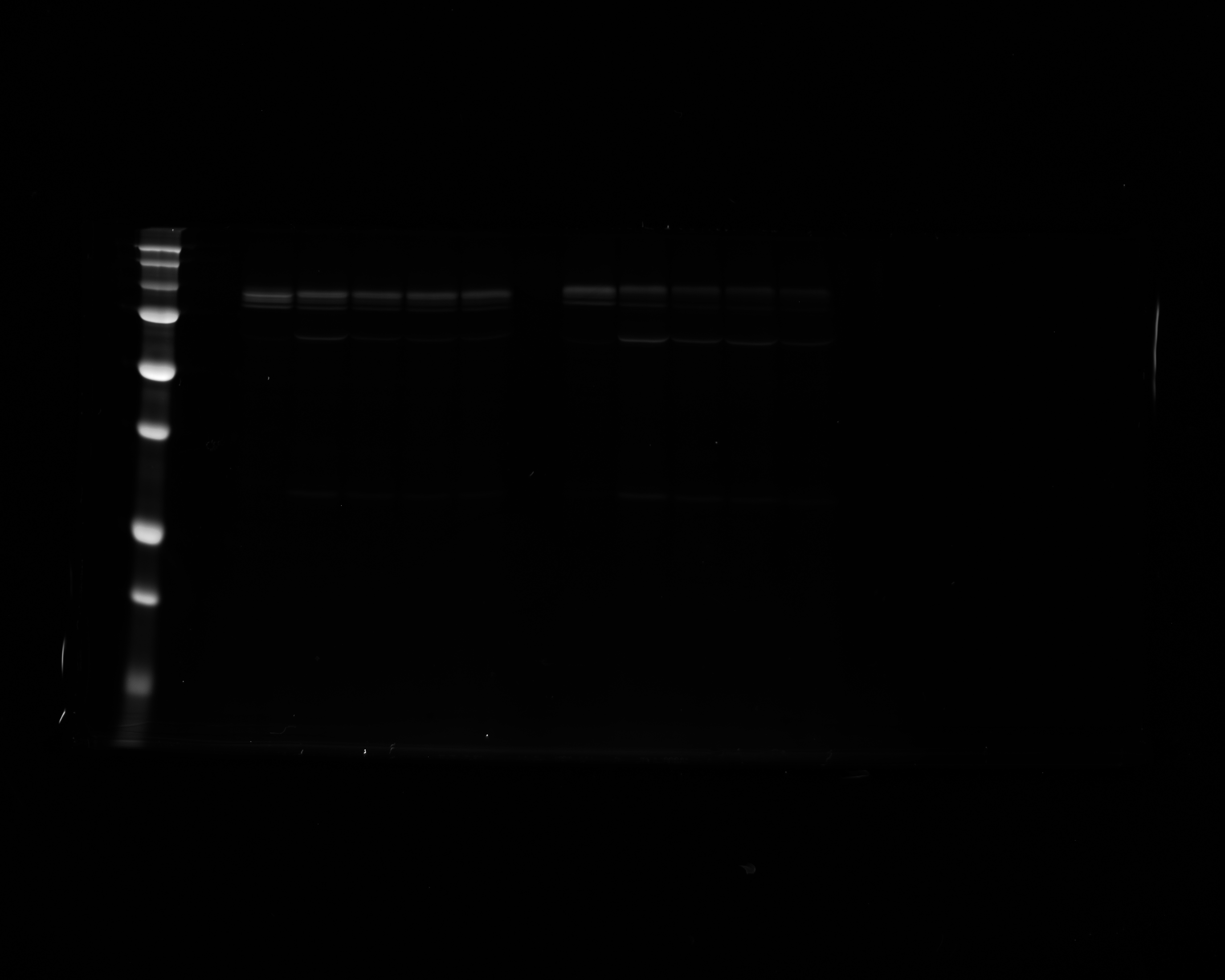

Supplement: Source data 1. [file elife-67261-data1.zip › Source data files/Raw blot images/Figure 6/Figure 6e in-gel far red fluorescence.tif]

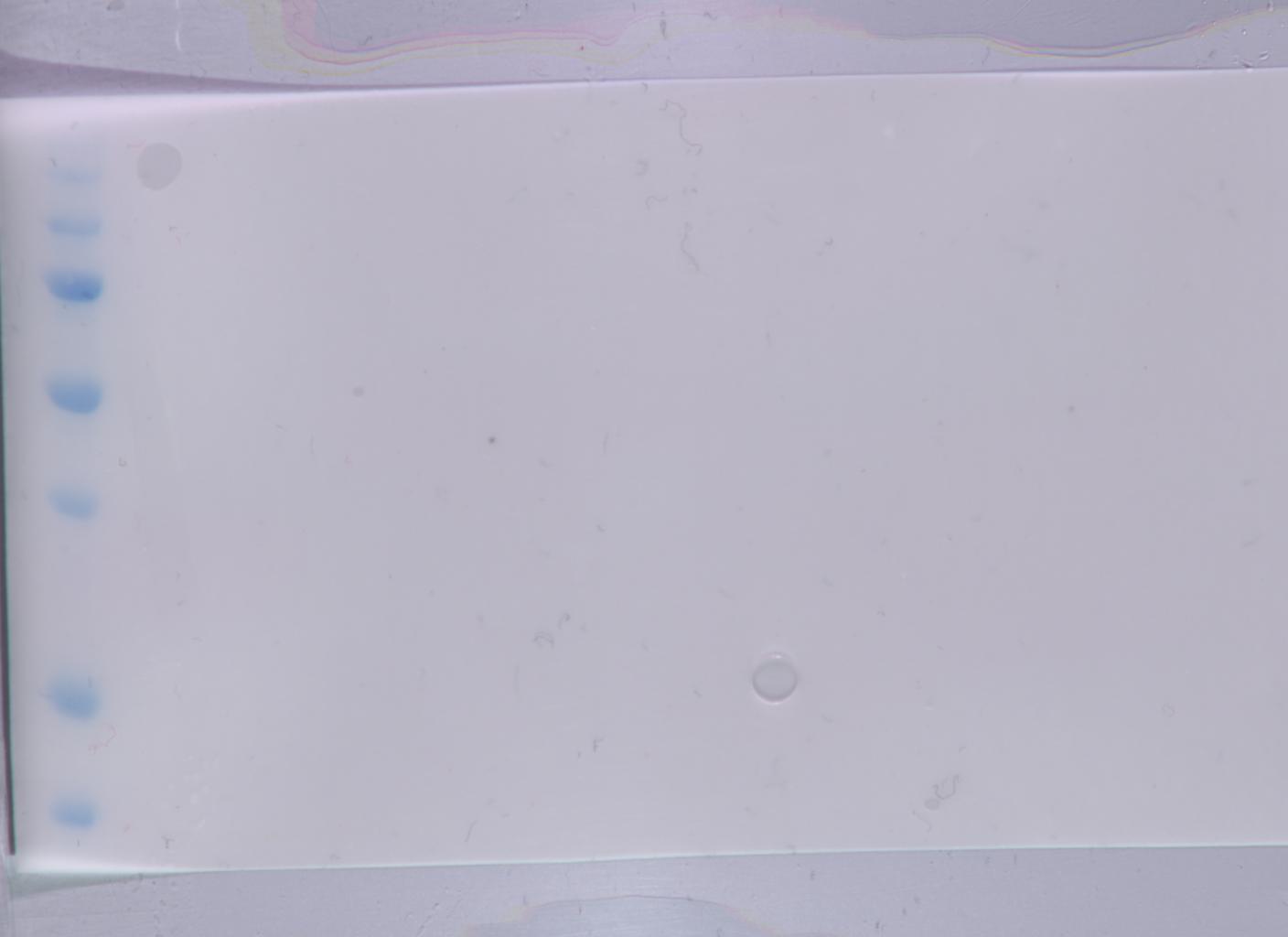

Supplement: Source data 1. [file elife-67261-data1.zip › Source data files/Raw blot images/Figure 7 - Figure Supplement 1/Figure 7 - Figure Supplement 1g anti-SYT7 Ladder.jpg]

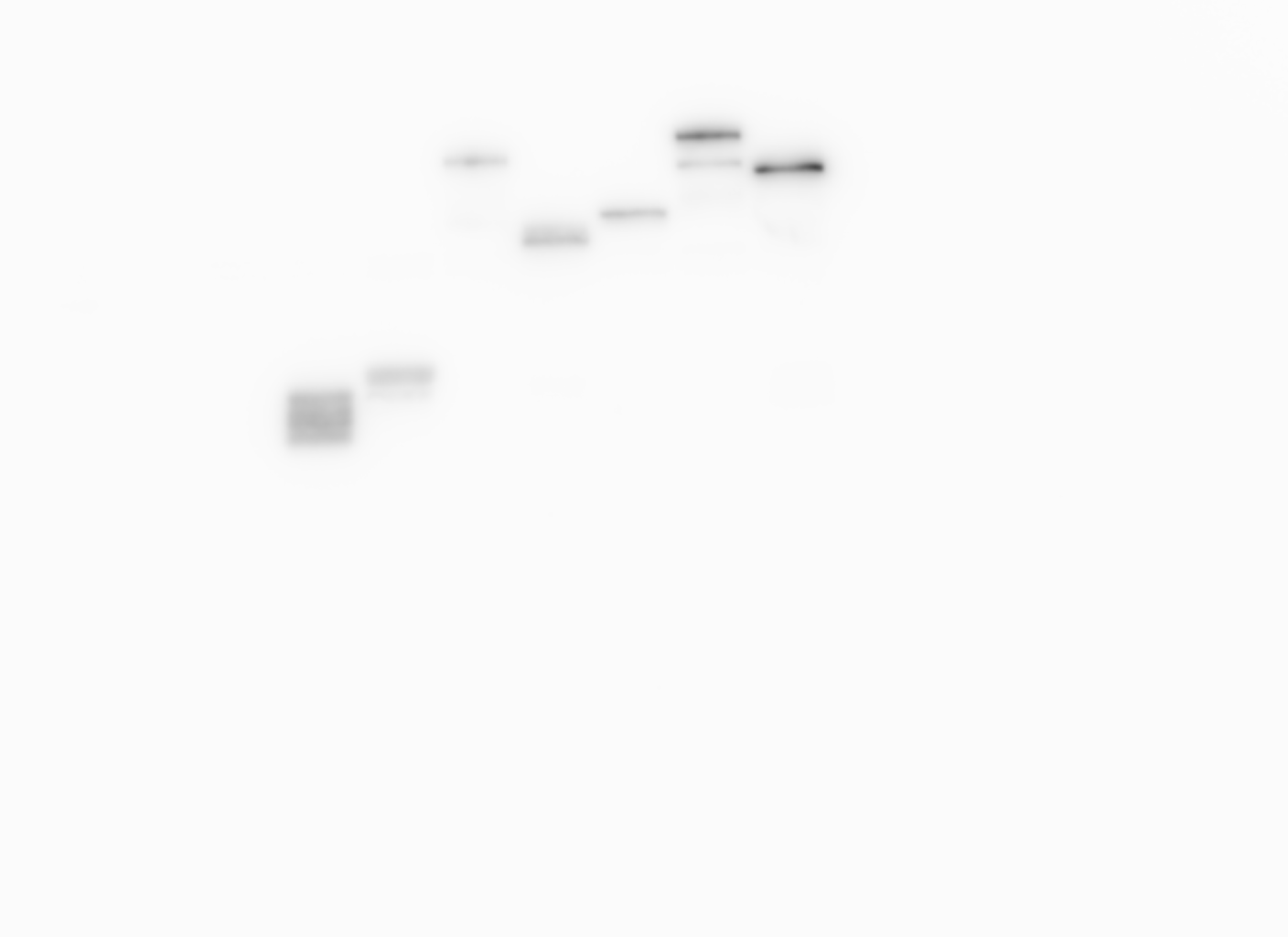

Supplement: Source data 1. [file elife-67261-data1.zip › Source data files/Raw blot images/Figure 7 - Figure Supplement 1/Figure 7 - Figure Supplement 1g anti-SYT7.tif]
